# Design and Synthesis of Novel Antioxidant 2-Substituted-5,7,8-Trimethyl-1,4-Benzoxazine Hybrids: Effects on Young and Senescent Fibroblasts

**DOI:** 10.3390/antiox13070798

**Published:** 2024-06-29

**Authors:** Theano Fotopoulou, Adamantia Papadopoulou, Andromachi Tzani, Michail Mamais, Eleni Mavrogonatou, Harris Pratsinis, Maria Koufaki, Dimitris Kletsas, Theodora Calogeropoulou

**Affiliations:** 1 Institute of Chemical Biology, National Hellenic Research Foundation, 48 Vassileos Constantinou Avenue, 11635 Athens, Greece; tfotop@eie.gr (T.F.); andromachi.tzani@gmail.com (A.T.); mmamais@eie.gr (M.M.); mkoufa@eie.gr (M.K.); 2 Institute of Biosciences & Applications, NCSR “Demokritos”, T. Patriarchou Grigoriou & Neapoleos, 15310 Athens, Greece; apapad@bio.demokritos.gr (A.P.); elmavro@bio.demokritos.gr (E.M.); hprats@bio.demokritos.gr (H.P.)

**Keywords:** antioxidant, anti-ageing, 1,4-benzoxazines, catechol, resorcinol, hybrids, young and senescent skin fibroblasts, *heme oxygenase-1* gene, GSH enhancers

## Abstract

The exponential growth of the aged population worldwide is followed by an increase in the prevalence of age-related disorders. Oxidative stress plays central role in damage accumulation during ageing and cell senescence. Thus, a major target of today’s anti-ageing research has been focused on antioxidants counteracting senescence. In the current work, six novel 5,7,8-trimethyl-1,4-benzoxazine/catechol or resorcinol hybrids were synthesized connected through a methoxymethyl-1,2,3-triazolyl or a 1,2,3-triazoly linker. The compounds were evaluated for their antioxidant capacity in a cell-free system and for their ability to reduce intracellular ROS levels in human skin fibroblasts, both young (early-passage) and senescent. The most efficient compounds were further tested in these cells for their ability to induce the expression of the gene *heme oxygenase-1* (*ho-1*), known to regulate redox homeostasis, and cellular glutathione (GSH) levels. Overall, the two catechol derivatives were found to be more potent than the resorcinol analogues. Furthermore, these two derivatives were shown to act coordinately as radical scavengers, ROS inhibitors, *ho-1* gene expression inducers, and GSH enhancers. Interestingly, one of the two catechol derivatives was also found to enhance human skin fibroblast viability. The properties of the synthesized compounds support their potential use in cosmetic applications, especially in products targeting skin ageing.

## 1. Introduction

The aged population worldwide is increasing exponentially, with estimations by the United Nations that one out of six people will be over 65 years old in 2050 [1]. One of the main consequences is the amplified prevalence of age-related disorders, such as cardiovascular diseases, cancer, musculoskeletal syndromes, and neurodegenerative diseases [2]. According to the “free radical theory of ageing”, proposed already in 1956 [3], oxidative stress is the major factor leading to age-related accumulation of defects. Observations at the cellular level support the idea that free radicals and reactive oxygen species (ROS) may lead to additive damages of subcellular organelles, especially mitochondria, thus creating further generation of more free radicals and a positive feedback loop [4]. Moreover, ROS and free radicals have been shown to induce cellular senescence to many different cell types [5,6,7]. Senescent cells are characterized by their inability to proliferate, by their pro-inflammatory and catabolic phenotype, and by their involvement in the pathogenesis and/or aggravation of age-related diseases [8]. Consequently, cellular senescence has been included among the “Hallmarks of Ageing” [9]. Accordingly, a major target of today’s anti-ageing research has been focused on antioxidants counteracting senescence [10].

**Figure 1:**
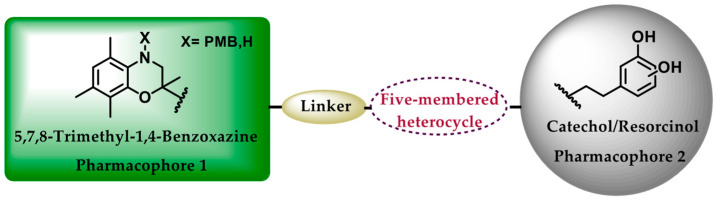
Design of the compounds of the present study.

1,2-, 1,3-, and 1,4-benzoxazines are considered as one of the key classes of organic molecules endowed with a broad spectrum of biological activities [11,12]. More specifically, 1,4-benzoxazines represent a privileged scaffold in drug discovery due to their fascinating pharmacological profile [13,14]. They have been investigated as potential antimicrobial/antifungal [15,16,17,18,19,20], antioxidant [21,22,23,24,25,26], anti-infective [27], antidiabetic [28], or anticancer agents [29,30,31,32,33,34,35], as well as against neurodegenerative [36] and cardiovascular disorders [37,38]. In particular, the 5,7,8-trimethyl-1,4-benzoxazine moiety can be considered as a bioisostere of the 5,7,8-trimethyl-1,4-benzopyran nucleus [39,40] that is the key heterocycle moiety of the well-known chain-breaking antioxidant vitamin E. Calogeropoulou et al. were the first to synthesize a number of 5,7,8-trimethyl-1,4-benzoxazine derivatives endowed with an array of biological activities, specifically against arrhythmias associated with ischemia-reperfusion injury [41], against toxoplasmosis inhibiting the proliferation of *Toxoplasma gondii* tachyzoites [27], against prion diseases inhibiting the formation of PrPSc [42], and finally as modulators of the AtoSC two-component system mediated signaling [43]. Thus, as a continuation of our previous work, herein we employed the well-established medicinal chemistry approach of molecular hybridization [44] and synthesized six compounds combining the 5,7,8-trimethyl-1,4-benzoxazine scaffold and catechol or resorcinol moieties. The two pharmacophores are connected through the amide bond bioisostere of the 1,2,3-triazole heterocycle (Figure 1) [45].

1,2,3-triazoles have been employed as linkers in the synthesis of hybrid compounds due to their sturdiness against oxidation or reduction and their increased solubility and improved interactions with biological targets due to their ability to form hydrogen bonds. Moreover, phenolic compounds such as catechol or resorcinol derivatives are active against ageing and age-related diseases and disorders, mainly due to their potent antioxidant profile. For example, one of the most well-known catechol derivatives, hydroxytyrosol (isolated from extra virgin oil), possesses powerful antioxidant, anti-inflammatory [46,47,48], wound healing [49], neuroprotective [50,51], anti-ageing [52,53], and cardioprotective properties [54,55]. In turn, resorcinol-substituted compounds possess activity against hyperpigmentation [56,57,58], reactive oxygen species (ROS) [59], or as cholinesterase inhibitors [60]. The new compounds were tested for their antioxidant activities in cell-free and cell-based systems. In particular, they were initially evaluated for their antioxidant capacity in a cell-free system and their ability to reduce intracellular ROS levels using human young (early-passage) and senescent skin fibroblasts. Furthermore, compounds possessing intracellular antioxidant properties were tested for their ability to induce the expression of gene *heme oxygenase-1* (*ho-1*), known to regulate redox homeostasis, in young and senescent human skin fibroblasts. Finally, their effect on cellular glutathione (GSH) levels in human skin fibroblasts was assessed.

## 2. Materials and Methods

### 2.1. Chemistry

#### 2.1.1. General

Melting points were determined with an Electrothermal Digital Melting Point Apparatus, Cole-Parmer ET0001/Version 1.0 and are uncorrected. ^1^H NMR spectra were recorded on Varian spectrometers (Varian Medical Systems, Inc., Palo Alto, CA, USA) operating at 300 or 600 MHz. ^13^C NMR spectra were recorded at 75 or 150 MHz, using CDCl_3_, CD_3_OD or (CD_3_)_2_CO as solvents. Chemical shifts are reported in *δ* units, parts per million (ppm) downfield from TMS. Electro-spray ionization (ESI) mass spectra were recorded on a LC-MSn Fleet Thermo spectrometer (Thermo Fisher Scientific, Waltham, MA, USA) using MeOH as solvent. HRMS spectra were recorded in the ESI or APCI mode on a UPLC-MSn Orbitrap Velos-Thermo spectrometer (Thermo Fisher Scientific, Waltham, MA, USA). Reactions under microwave irradiation were performed in a CEM Discover Lab Mate reactor (CEM Corporation, Matthews, NC, USA). Flash column chromatography (FCC) was performed on Merck silica gel 60 (230–400 mesh) Merck KGaA, Darmstadt, Germany) and TLC on Merck 60 F254 films (0.2 mm) precoated glass plates Merck KGaA, Darmstadt, Germany). Spots were visualized with UV light at 254 nm and PMA stain (phosphomolybdic acid 10% in absolute ethanol). All solvents were dried and/or purified according to standard procedures prior to use. All reagents employed in the present work were purchased from commercial suppliers and used without further purification. Reactions were run in flame-dried glassware under an atmosphere of argon.

#### 2.1.2. Synthetic Procedures

##### Ethyl 4-(4-methoxybenzyl)-2,5,7,8-tetramethyl-3-oxo-3,4-dihydro-2H-benzo[*b*][1,4]oxazine-2-carboxylate (**4**)

To a solution of ethyl 2,5,7,8-tetramethyl-3-oxo-3,4-dihydro-2*H*-benzo[*b*][1,4]oxazine-2-carboxylate (**3**) [0.33 g, 1.20 mmol, (synthesized as described in the Supplementary Material)] in toluene (15 mL), a solution of K_2_CO_3_ (0.74 g, 3.60 mmol) in H_2_O (1.67 mL), tetrabutylammonium bromide (TBAB) (0.08 g, 0.24 mmol) and *p*-methoxybenzyl chloride (PMBCl) (0.47 g, 3.01 mmol) were added. The resulting suspension was refluxed at 80 °C for 19 h. Then, the solvent was evaporated in vacuo and the residue was purified by FCC (CH_2_Cl_2_/MeOH 99:1 *v*/*v*) to afford ethyl 4-(4-methoxybenzyl)-2,5,7,8-tetramethyl-3-oxo-3,4-dihydro-2*H*-benzo[*b*][1,4]oxazine-2-carboxylate (**4**) as a colorless oil (0.33 g, 69% yield). ^1^H NMR (600 MHz, CDCl_3_): *δ* 7.06 (d, *J* = 8.4 Hz, 2H), 6.78 (d, *J* = 8.5 Hz, 2H), 6.58 (s, 1H), 5.22 (d, *J* = 15.9 Hz, 1H), 4.92 (d, *J* = 15.9 Hz, 1H), 4.06 (q, *J* = 7.1 Hz, 2H), 3.75 (s, 3H), 2.21 (s, 3H), 2.18 (s, 3H), 2.17 (s, 3H), 1.86 (s, 3H), 1.03 (t, *J* = 7.1 Hz, 3H); ^13^C NMR (150 MHz, CDCl_3_): *δ* 169.5, 167.0, 158.6, 145.9, 133.8, 129.3, 128.0, 127.9, 127.6, 126.7, 124.5, 123.9, 114.0, 81.9, 61.8, 55.3, 48.8, 21.0, 20.7, 14.0, 11.8; ESI-HRMS (*m*/*z*): calcd for C_23_H_27_O_5_NNa [Μ + Na]^+^ 420.1781; found 420.1772.

##### (4-(4-Methoxybenzyl)-2,5,7,8-tetramethyl-3,4-dihydro-2*H*-benzo[*b*][1,4]oxazin-2-yl)methanol (**5**)

To a solution of ethyl 4-(4-methoxybenzyl)-2,5,7,8-tetramethyl-3-oxo-3,4-dihydro-2*H*-benzo[*b*][1,4]oxazine-2-carboxylate (**4**) (0.55 g, 1.38 mmol) in THF (25 mL), (CH_3_)_2_SBH_3_ (0.44 mL, 4.66 mmol) was added dropwise at 0 °C and the reaction was stirred at the same temperature for 5 min, then at 67 °C for 16 h. Upon completion, the reaction mixture was cooled to 0 °C, quenched with methanol (2ml) and stirred for a further 10 min. The solvents were evaporated in vacuo and the residue was extracted with ethyl acetate. The organic layer was washed with saturated NaHCO_3_, 1 N NaHSO_4_, brine, dried over Na_2_SO_4_, filtered, and concentrated under reduced pressure. The oily residue was purified by FCC (CH_2_Cl_2_/acetone 99:1 *v*/*v*) to afford (4-(4-methoxybenzyl)-2,5,7,8-tetramethyl-3,4-dihydro-2*H*-benzo[*b*][1,4]oxazin-2-yl)methanol (**5**) as a white solid (0.411 g, 87% yield). ^1^H NMR (600 MHz, CD_3_OD): *δ* 7.38 (d, *J* = 8.6 Hz, 2H), 6.90 (d, *J* = 8.7 Hz, 2H), 6.49 (s, 1H), 4.31, 4.27 (ABq, *J* = 15.7 Hz, 2H), 3.78 (s, 3H), 3.55 (d, *J* = 11.1 Hz, 1H), 3.50 (d, *J* = 11.1 Hz, 1H), 3.14, (d, *J* = 13.7 Hz, 1H), 2.84 (d, *J* = 13.7 Hz, 1H), 2.17 (s, 3H), 2.13 (s, 3H), 2.05 (s, 2H), 1.29 (s, 3H); ^13^C NMR (75 MHz, CD_3_OD): *δ* 160.2, 145.2, 132.8, 132.6, 130.2, 129.6, 127.3, 125.5, 123.0, 114.8, 75.6, 66.6, 56.0, 55.7, 22.2, 19.6, 19.5, 11.8; ESI-HRMS (*m*/*z*): calcd for C_21_H_27_O_3_NNa [Μ + Na]^+^ 364.1883; found 364.1876.

##### 4-(4-Methoxybenzyl)-2,5,7,8-tetramethyl-3,4-dihydro-2*H*-benzo[*b*][1,4]oxazine-2-carbaldehyde (**6**)

Anhydrous DMSO (226 μL, 3.18 mmol) was added to (4-(4-methoxybenzyl)-2,5,7,8-tetramethyl-3,4-dihydro-2*H*-benzo[*b*][1,4]oxazin-2-yl)methanol (**5**) (0.18 g, 0.53 mmol), followed by the addition of *N,N*-diisopropylcarbodiimide (248 μL, 1.60 mmol) and Cl_2_CHCOOH (26 μL, 0.32 mmol). The resulting mixture was stirred at room temperature for 2 h. After completion of the reaction, the mixture was extracted with Et_2_O, and the organic layer was washed with saturated aqueous NaCl, dried with Na_2_SO_4_, filtered, and the solvent evaporated in vacuo. Purification by FCC (CHCl_3_, 100% *v*/*v*) afforded 4-(4-methoxybenzyl)-2,5,7,8-tetramethyl-3,4-dihydro-2H-benzo[*b*][1,4]oxazine-2-carbaldehyde (**6**) as a white solid (0.148 g, 82%). ^1^H NMR (600 MHz, CDCl_3_): *δ* 9.91 (s, 1H), 7.33 (d, *J* = 8.6 Hz, 2H), 6.91 (d, *J* = 8.6 Hz, 2H), 6.66 (s, 1H), 4.20 (d, *J* = 15.0 Hz, 1H), 3.83 (s, 3H), 3.52 (d, *J* = 15.1 Hz, 1H), 3.42 (d, *J* = 14.3 Hz, 1H), 2.80 (d, *J* = 14.2 Hz, 1H), 2.29 (s, 3H), 2.24 (s, 3H), 2.21 (s, 3H), 1.35 (s, 3H); ^13^C NMR (150 MHz, CDCl_3_): *δ* 204.3, 159.2, 144.7, 131.6, 131.0, 129.9, 129.3, 128.6, 124.9, 122.5, 114.1, 80.8, 58.4, 55.4, 51.3, 21.8, 19.7, 18.1, 11.9; APCI-HRMS (*m*/*z*): calcd for C_21_H_26_O_3_N [Μ + H]^+^ 340.1907; found 340.1905.

##### 2-Ethynyl-4-(4-methoxybenzyl)-2,5,7,8-tetramethyl-3,4-dihydro-2*H*-benzo[*b*][1,4]oxazine (**7**)

To a solution of 4-(4-methoxybenzyl)-2,5,7,8-tetramethyl-3,4-dihydro-2*H*-benzo[*b*][1,4]oxazine-2-carbaldehyde (**6**) (0.10 g, 0.29 mmol) in 12 mL dry CH_3_OH was added at 0 °C K_2_CO_3_ (0.08 g, 0.58 mmol) followed by the Bestmann–Ohira reagent (0.11 g, 0.58 mmol). The reaction mixture was warmed at ambient temperature and stirred for 2.5 h. The solvent was then evaporated and the residue was extracted with Et_2_O and saturated aqueous NaHCO_3_. The organic layer was washed with brine, dried over Na_2_SO_4_, filtered, and the solvent was evaporated in vacuo to afford 2-ethynyl-4-(4-methoxybenzyl)-2,5,7,8-tetramethyl-3,4-dihydro-2*H*-benzo[*b*][1,4]oxazine (**7**). ^1^H NMR (600 MHz, acetone-*d_6_*): *δ* 7.44 (d, *J* = 8.7 Hz, 2H), 6.96 (d, *J* = 8.7 Hz, 2H), 6.58 (s, 1H), 4.92 (d, *J* = 15.6 Hz, 1H), 4.26 (d, *J* = 15.6 Hz, 1H), 3.81 (s, 3H), 3.38 (d, *J* = 14.1 Hz, 1H), 3.02 (s, 1H) 2.83 (d, *J* = 14.1 Hz, 1H), 2.22 (s, 3H), 2.16 (s, 3H), 2.08 (s, 3H), 1.57 (s, 3H); ^13^C NMR (150 MHz acetone-*d_6_*): *δ* 159.9, 144.6, 131.9, 131.7, 130.5, 129.7, 128.0, 125.6, 122.4, 114.7, 86.3, 74.5, 66.9, 57.7, 55.5, 54.8, 27.9, 19.5, 18.8, 11.9; APCI-HRMS (*m*/*z*): calcd for C_22_H_26_O_2_N [Μ + H]^+^ 336.1958; found 336.1956.

##### 4-(4-Μethoxybenzyl)-2,5,7,8-tetramethyl-2-((prop-2-yn-1-yloxy)methyl)-3,4-dihydro-2*H*-benzo[*b*][1,4]oxazine (**8**)

To a solution of (4-(4-methoxybenzyl)-2,5,7,8-tetramethyl-3,4-dihydro-2*H*-benzo[*b*][1,4]oxazin-2-yl)methanol (**5**) (0.14 g, 0.43 mmol) in dry DMF (10 mL), NaH (0.05 g, 2.15 mmol, 60% in oil) was added at 0 °C and the mixture was stirred at ambient temperature for 1 h. Then, propargyl bromide (0.26 g, 2.15 mmol, 80% in toluene) was added dropwise at 0 °C and it was stirred at room temperature overnight. The reaction was quenched with water and was extracted with ethyl acetate. The organic layer was washed with brine, dried over Na_2_SO_4_, and concentrated in vacuo. The crude residue was purified by FCC (hexanes/EtOAc 90:10 *v*/*v*) to give 4-(4-μethoxybenzyl)-2,5,7,8-tetramethyl-2-((prop-2-yn-1-yloxy)methyl)-3,4-dihydro-2*H*-benzo[*b*][1,4]oxazine (**8**) as a yellow oil (0.114 g, 70% yield). ^1^H NMR (300 MHz, CDCl_3_): *δ* 7.42 (d, *J* = 8.3 Hz, 2H), 6.92 (d, *J* = 8.6 Hz, 2H), 6.58 (s, 1H), 4.42–4.26 (m, 2H), 4.20 (dd, *J* = 8.8 and 2.4 Hz, 2H), 3.83 (s, 3H), 3.68 (d, *J* = 9.2 Hz, 1H), 3.42 (d, *J* = 9.2 Hz, 1H), 3.19 (d, *J* = 14.0 Hz, 1H), 2.83 (d, *J* = 14.0 Hz, 1H), 2.43 (t, *J* = 2.4 Hz, 1H), 2.22 (s, 3H), 2.19 (s, 3H), 2.10 (s, 3H), 1.34 (s, 3H); ^13^C NMR (75 MHz CDCl_3_): *δ* 158.8, 144.0, 131.8, 131.2, 129.6, 128.6, 126.8, 124.6, 122.3, 114.0, 79.6, 74.9, 73.8, 72.8, 58.6, 57.7, 55.5, 50.8, 23.1, 19.6, 19.2, 11.8; APCI-HRMS (*m*/*z*): calcd for C_24_H_30_O_3_N [Μ + H]^+^ 380.2220; found 380.2211.

##### Synthesis of 1,4-disubstituted 1,2,3-triazoles (**19**, **20**, **21**, **23**, **25**)

**Method A.** To a solution of 2-ethynyl-4-(4-methoxybenzyl)-2,5,7,8-tetramethyl-3,4-dihydro-2*H*-benzo[*b*][1,4]oxazine (**7**) (1eq) in a mixture of CH_2_Cl_2_/H_2_O (1:1), the appropriate azide (1.1 eq), CuSO_4_·5H_2_O (0.3 eq), and sodium ascorbate (0.6 eq), were added, and the reaction mixture was stirred at room temperature overnight. Then, it was diluted with CH_2_Cl_2_ and the organic layer was washed with saturated aqueous NH_4_Cl, NaCl, dried over Na_2_SO_4_, filtered, and the solvent was evaporated in vacuo. The desired product was obtained after FCC.

**Method B.** To a solution of 2-ethynyl-4-(4-methoxybenzyl)-2,5,7,8-tetramethyl-3,4-dihydro-2*H*-benzo[*b*][1,4]oxazine (**7**) or 4-(4-methoxybenzyl)-2,5,7,8-tetramethyl-2-((prop-2-yn-1-yloxy)methyl)-3,4-dihydro-2*H*-benzo[*b*][1,4]oxazine (**8**) (1eq) in a mixture of *t*-BuOH/H_2_O (1:1), the appropriate azide (1.1 eq), CuSO_4_·5H_2_O (0.3 eq), and sodium ascorbate (0.6 eq), were added. The reaction was microwave irradiated (80 W, 90 °C, 30 min). Upon completion, the mixture was diluted with ethyl acetate. The organic layer was washed with saturated aqueous NH_4_Cl, NaCl, dried over Na_2_SO_4_, filtered, and the solvent was evaporated in vacuo. The desired product was obtained after FCC (hexanes/EtOAc, 70:30 to 50:50 *v*/*v*).

**4-(2-(4-(4-(4-methoxybenzyl)-2,5,7,8-tetramethyl-3,4-dihydro-2H-benzo[*b*][1,4]oxazin-2-yl)-1H-1,2,3-triazol-1-yl)ethyl)benzene-1,2-diol (19, TC488)**

Following Method A, using 2-ethynyl-4-(4-methoxybenzyl)-2,5,7,8-tetramethyl-3,4-dihydro-2*H*-benzo[*b*][1,4]oxazine (**7**) (0.06 g, 0.17 mmol) and 4-(2-azidoethyl)benzene-1,2-diol (**14**) [0.03 g, 0.19 mmol, (synthesized as described in the Supplementary Material)] in 5 mL CH_2_Cl_2_/H_2_O (1:1), compound 4-(2-(4-(4-(4-methoxybenzyl)-2,5,7,8-tetramethyl-3,4-dihydro-2H-benzo[*b*][1,4]oxazin-2-yl)-1H-1,2,3-triazol-1-yl)ethyl)benzene-1,2-diol (**19**, **TC488**) was obtained after FCC (CH_2_Cl_2_/EtOAc 85:15 *v*/*v*) as a green oil (0.050 g, 57%). ^1^H NMR (600 MHz, CDCl_3_): *δ* 7.90 (brs, 1H, -OH), 7.21 (d, *J* = 8.2 Hz, 2H), 7.16 (s, 1H), 6.95 (s, 1H), 6.77 (d, *J* = 8.4 Hz, 2H), 6.73 (d, *J* = 8.0 Hz, 1H), 6.62 (s, 1H), 5.76 (brs, 1H, -OH), 4.61–4.48 (m, 2H), 3.97 (d, *J* = 15.1 Hz, 1H), 3.76 (s, 3H), 3.60 (d, *J* = 14.2 Hz, 1H), 3.16 (d, *J*= 15.1 Hz, 1H), 3.09–3.05 (m, 3H), 2.24 (s, 3H), 2.23 (s, 3H), 2.19 (s, 3H), 1.62 (s, 3H) (Figure S1); ^13^C NMR (75 MHz, CDCl_3_): *δ* 158.9, 151.9, 144.5, 144.2, 143.6, 129.3, 128.8, 127.6, 124.9, 121.9, 120.5, 115.8, 115.2, 113.9, 72.4, 60.6, 56.7, 55.4, 52.3, 36.5, 27.2, 19.6, 18.81, 11.89 (Figure S2); ESI-HRMS (*m*/*z*): calcd. for C_30_H_35_O_4_N_4_ [Μ + H]^+^ 515.2653; found 515.2647 (Figure S3).

**2-(1-(3,5-dimethoxyphenethyl)-1H-1,2,3-triazol-4-yl)-4-(4-methoxybenzyl)-2,5,7,8-tetramethyl-3,4-dihydro-2H-benzo[*b*][1,4]oxazine (20)**

Following Method A, using 2-ethynyl-4-(4-methoxybenzyl)-2,5,7,8-tetramethyl-3,4-dihydro-2*H*-benzo[*b*][1,4]oxazine (**7**) (0.03 g, 0.09 mmol) and 1-(2-azidoethyl)-3,5-dimethoxybenzene (**17**) [0.02 g, 0.10 mmol, (synthesized as described in the Supplementary Material)] in 3 mL CH_2_Cl_2_/H_2_O (1:1) compound 2-(1-(3,5-dimethoxyphenethyl)-1*H*-1,2,3-triazol-4-yl)-4-(4-methoxybenzyl)-2,5,7,8-tetramethyl-3,4-dihydro-2H-benzo[*b*][1,4]oxazine (**20**) was obtained after FCC (petroleum ether/EtOAc 80:20 to 70:30 *v*/*v*) as a white foam (0.032 g, 67%). ^1^H NMR (600 MHz, CDCl_3_): *δ* 7.25 (d, *J* = 8.1 Hz, 2H), 7.05 (s, 1H), 6.88 (d, *J* = 8.1 Hz, 2H), 6.60 (s, 1H), 6.30 (d, *J* = 2.3 Hz, 1H), 6.22 (s, 2H), 4.53 (ddt, *J* = 62.9, 14.2 and 7.2 Hz, 2H), 3.92 (d, *J* = 15.2 Hz, 1H), 3.81 (s, 3H), 3.70 (s, 6H), 3.61 (d, *J* = 14.1 Hz, 1H), 3.23–2.99 (m, 4H), 2.23 (s, 3H), 2.21 (s, 3H), 2.15 (s, 3H), 1.62 (s, 3H); ^13^C NMR (75 MHz, CDCl_3_): *δ* 161.2, 158.8, 151.9, 144.2, 139.2, 131.0, 129.9, 129.2, 127.5, 124.7, 121.9, 121.5, 113.9, 106.8, 99.0, 72.5, 56.5, 55.4, 53.4, 51.3, 37.1, 27.1, 19.6, 18.8, 11.8; APCI-HRMS (*m*/*z*): calcd. for C_32_H_39_O_4_N_4_ [Μ + H]^+^ 543.2966; found 543.2966.

**5-(2-(4-(4-(4-methoxybenzyl)-2,5,7,8-tetramethyl-3,4-dihydro-2H-benzo[*b*][1,4]oxazin-2-yl)-1H-1,2,3-triazol-1-yl)ethyl)benzene-1,3-diol (21, TC489)**

Following Method B, using 2-ethynyl-4-(4-methoxybenzyl)-2,5,7,8-tetramethyl-3,4-dihydro-2*H*-benzo[*b*][1,4]oxazine (**7**) (0.02 g, 0.06 mmol) and 5-(2-azidoethyl)benzene-1,3-diol (**18**) [0.01 g, 0.07 mmol, (synthesized as described in the Supplementary Material)] in 2 mL CH_2_Cl_2_/H_2_O (1:1) 5-(2-(4-(4-(4-methoxybenzyl)-2,5,7,8-tetramethyl-3,4-dihydro-2*H*-benzo[*b*][1,4]oxazin-2-yl)-1*H*-1,2,3-triazol-1-yl)ethyl)benzene-1,3-diol (**21, TC489**) was obtained after FCC (CH_2_Cl_2_/acetone 88:12 *v*/*v*) as an off-white solid (0.024 g, 67%). ^1^H NMR (600 MHz, CD_3_OD): *δ* 7.23 (s, 1H), 7.16 (d, *J* = 8.4 Hz, 2H), 6.86 (d, *J* = 8.6 Hz, 2H), 6.54 (s, 1H), 6.04 (s, 1H), 6.02 (s, 2H), 4.59 (t, *J* = 6.9 Hz, 2H), 3.87 (d, *J* = 15.6 Hz, 1H), 3.78 (s, 3H), 3.50 (d, *J* = 14.0 Hz, 1H), 3.07–2.87 (m, 4H), 2.20 (s, 3H), 2.15 (s, 3H), 2.14 (s, 3H), 1.56 (s, 3H) (Figure S4); ^13^C NMR (150 MHz, CD_3_OD): *δ* 160.2, 159.7, 152.6, 145.2, 140.4, 132.2, 131.9, 130.9, 129.9, 128.3, 125.8, 123.6, 122.2, 114.7, 108.1, 102.1, 73.0, 57.5, 55.7, 54.9, 52.1, 37.3, 27.3, 19.6, 18.9, 11.9 (Figure S5); ESI-HRMS (*m*/*z*): calcd. for C_30_H_35_O_4_N_4_ [Μ + H]^+^ 515.2653; found 515.2653 (Figure S6).

**2-(((1-(2-(2,2-dimethylbenzo[*d*][1,3]dioxol-5-yl)ethyl)-1H-1,2,3-triazol-4-yl)methoxy)methyl)-4-(4-methoxybenzyl)-2,5,7,8-tetramethyl-3,4-dihydro-2*H*-benzo[*b*][1,4]oxazine (23)**

Following Method B, using 4-(4-methoxybenzyl)-2,5,7,8-tetramethyl-2-((prop-2-yn-1-yloxy)methyl)-3,4-dihydro-2*H*-benzo[*b*][1,4]oxazine (**8**) (0.05 g, 0.13 mmol) in 1.5 mL *t*-BuOH/H_2_O (1:1) and 5-(2-azidoethyl)-2,2-dimethylbenzo[*d*][1,3]dioxole (**13**) [0.03 g, 0.14 mmol, (synthesized as described in the Supplementary Material)] compound 2-(((1-(2-(2,2-dimethylbenzo[*d*][1,3]dioxol-5-yl)ethyl)-1H-1,2,3-triazol-4-yl)methoxy)methyl)-4-(4-methoxybenzyl)-2,5,7,8-tetramethyl-3,4-dihydro-2*H*-benzo[*b*][1,4]oxazine (**23**) was obtained as white solid (0.061 g, 76%). ^1^H NMR (600 MHz, acetone-*d_6_*): *δ* 7.75 (s, 1H), 7.42 (d, *J* = 8.5 Hz, 2H), 6.92 (d, *J* = 8.5 Hz, 2H), 6.66 (d, *J* = 1.7 Hz, 1H), 6.58–6.49 (m, 3H), 4.65, 4.62 (ABq, *J*_AB_ = 12.3 Hz, 2H), 4.58 (td, *J* = 7.2 and 1.5 Hz, 2H), 4.28 (d, *J* = 15.8 Hz, 1H), 4.18 (d, *J* = 15.8 Hz, 1H), 3.79 (s, 3H), 3.57 (d, *J* = 9.2 Hz, 1H), 3.40 (d, *J* = 9.2 Hz, 1H), 3.15 (d, *J* = 13.9 Hz, 1H), 3.10 (t, *J* = 7.4 Hz, 2H), 2.76 (d, *J* = 13.9 Hz, 1H), 2.17 (s, 3H), 2.14 (s, 3H), 2.06 (s, 3H), 1.61 (d, *J* = 2.3 Hz, 6H), 1.29 (s, 3H); ^13^C NMR (75 MHz, acetone-*d_6_*): *δ* 159.7, 148.5, 147.2, 144.8, 144.7, 132.5, 132.0, 131.7, 129.8, 129.4, 127.18, 125.2, 124.2, 122.3, 122.1, 118.6, 114.6, 109.7, 108.8, 74.2, 73.2, 65.1, 58.1, 55.5, 52.0, 51.5, 37.0, 25.9, 23.4, 19.5, 19.3, 11.9; APCI-HRMS (*m*/*z*): calcd for C_35_H_43_O_5_N_4_ [Μ + H]^+^ 599.3228; found 599.3221.

**2-(((1-(2-(2,2-dimethylbenzo[*d*][1,3]dioxol-5-yl)ethyl)-1H-1,2,3-triazol-4-yl)methoxy)methyl)-4-(4-methoxybenzyl)-2,5,7,8-tetramethyl-3,4-dihydro-2H-benzo[*b*][1,4]oxazine (25)**

Following Method B, using 4-(4-methoxybenzyl)-2,5,7,8-tetramethyl-2-((prop-2-yn-1-yloxy)methyl)-3,4-dihydro-2*H*-benzo[*b*][1,4]oxazine (**8**) (0.05 g, 0.13 mmol) in 1.5 mL *t*-BuOH/H_2_O (1:1) and 1-(2-azidoethyl)-3,5-dimethoxybenzene (**17**) [0.03 g, 0.14 mmol, (synthesized as described in the Supplementary Material)] compound 2-(((1-(2-(2,2-dimethylbenzo[*d*][1,3]dioxol-5-yl)ethyl)-1H-1,2,3-triazol-4-yl)methoxy)methyl)-4-(4-methoxy benzyl)-2,5,7,8-tetramethyl-3,4-dihydro-2H-benzo[*b*][1,4]oxazine (**25**) was obtained as a yellowish oil (0.074 g, 96%). ^1^H NMR (600 MHz, acetone-*d_6_*): *δ* 7.76 (s, 1H), 7.41 (d, *J* = 8.6 Hz, 2H), 6.92 (d, *J* = 8.7 Hz, 2H), 6.51 (s, 1H), 6.36 (d, *J* = 2.3 Hz, 2H), 6.32 (t, *J* = 2.3 Hz, 1H), 4.65–4.61 (m, 4H), 4.29 (d, *J* = 15.8 Hz, 1H), 4.18 (d, *J* = 15.8 Hz, 1H), 3.79 (s, 3H), 3.71 (s, 6H), 3.58 (d, *J* = 9.3 Hz, 1H), 3.41 (d, *J* = 9.3 Hz, 1H), 3.17–3.11 (m, 3H), 2.76 (d, *J* = 13.9 Hz, 1H), 2.17 (s, 3H), 2.13 (s, 3H), 2.04 (s, 3H), 1.29 (s, 3H); ^13^C NMR (150 MHz, acetone-*d_6_*): *δ* 162.0, 159.7, 144.9, 144.7, 140.9, 132.4, 132.0, 129.8, 129.4, 127.2, 125.2, 124.2, 122.3, 114.6, 107.5, 99.4, 74.2, 73.3, 65.1, 58.1, 55.5, 55.5, 51.6, 51.5, 37.4, 23.3, 19.5, 19.3, 11.9; ESI-HRMS (*m*/*z*): calcd for C_34_H_43_O_5_N_4_ [Μ + H]^+^ 587.3228; found 587.3242; calcd for C_34_H_42_O_5_N_4_Na [Μ + Na]^+^ 609.3047; found 609.3057.

##### 4-(2-(4-(((4-(4-Methoxybenzyl)-2,5,7,8-tetramethyl-3,4-dihydro-2H-benzo[*b*][1,4]oxazin-2-yl)methoxy)methyl)-1H-1,2,3-triazol-1-yl)ethyl)benzene-1,2-diol (**24**, **TC483**)

To an ice-cold solution of 2-(((1-(2-(2,2-dimethylbenzo[*d*][1,3]dioxol-5-yl)ethyl)-1H-1,2,3-triazol-4-yl)methoxy)methyl)-4-(4-methoxybenzyl)-2,5,7,8-tetramethyl-3,4-dihydro-2*H*-benzo[*b*][1,4]oxazine (**23**) (0.03 g, 0.05 mmol) in degassed CHCl_3_ (1 mL), TFA (0.27 mL) and H_2_O (0.05 mL) were added and the reaction was warmed at ambient temperature and stirred for 1.5 h. Upon completion of the reaction, the solvent was concentrated in vacuo and the residue was extracted with ethyl acetate. The organic layer was washed with saturated aqueous NaHCO_3_, NaCl, dried over anhydrous Na_2_SO_4_, filtered, and the solvent was evaporated to dryness under reduced pressure. The title compound **24** (**TC483**) was obtained as a white solid (0.022 g, 81% yield) after FCC purification (hexanes/acetone 70:30 *v*/*v*). ^1^H NMR (600 MHz, acetone-*d*_6_): *δ* 7.74 (s, 1H), 7.41 (d, *J* = 8.6 Hz, 2H), 6.92 (d, *J* = 8.6 Hz, 2H), 6.72–6.69 (m, 1H), 6.68 (s, 1H), 6.50 (s, 1H), 6.47 (dd, *J* = 8.0 and 2.1 Hz, 1H), 4.64, 4.61 (ABq, *J*_AB_ = 12.3 Hz, 2H), 4.55 (t, *J* = 7.4 Hz, 2H), 4.28 (d, *J* = 15.8 Hz, 1H), 4.18 (d, *J* = 15.8 Hz, 1H), 3.79 (s, 3H), 3.57 (d, *J* = 9.3 Hz, 1H), 3.41 (d, *J* = 9.3 Hz, 1H), 3.15 (d, *J* = 13.9 Hz, 1H), 3.03 (t, *J* = 7.4 Hz, 2H), 2.76 (d, *J* = 13.9 Hz, 1H), 2.16 (s, 3H), 2.13 (s, 3H), 2.04 (s, 3H), 1.30 (s, 3H) (Figure S7); ^13^C NMR (150 MHz, acetone-*d*_6_): *δ* 159.7, 146.0, 144.8, 144.7, 132.5, 132.0, 130.1, 129.8, 129.4, 127.1, 125.2, 124.2, 122.3, 120.9, 116.6, 116.1, 114.6, 74.2, 73.2, 65.1, 58.1, 55.5, 36.7, 23.4, 19.5, 19.3, 11.9 (Figure S8); ESI-HRMS (*m*/*z*): calcd for C_32_H_39_N_4_O_5_ [M + H]^+^ 559.2915; found 559.2924 (Figure S9).

##### General Procedure for the Deprotection of Methoxy Groups of Compounds **20** and **25**

To a solution of compound **20** or **25** (1 eq) in dry CH_2_Cl_2_ (0.05 Μ), BF_3_S(Me)_2_ (10 eq) was added at 0 °C and the reaction mixture was stirred at ambient temperature for 2–24 h. After completion of the reaction (checked by TLC), the solvent and excess reagent were evaporated under argon stream. The residue was extracted with ethyl acetate. The organic layer was washed with saturated aqueous NaCl, dried over Na_2_SO_4_, and the solvent was evaporated in vacuo. The desired product was afforded after purification by FCC (CH_2_Cl_2_/acetone 90:10 *v*/*v*).

**5-(2-(4-(2,5,7,8-tetramethyl-3,4-dihydro-2H-benzo[*b*][1,4]oxazin-2-yl)-1H-1,2,3-triazol-1-yl)ethyl)benzene-1,3-diol (22, TC490) and 5-(2-(4-(4-(4-hydroxybenzyl)-2,5,7,8-tetramethyl-3,4-dihydro-2H-benzo[*b*][1,4]oxazin-2-yl)-1H-1,2,3-triazol-1-yl)ethyl)benzene-1,3-diol (22a, TC491).**

The title compounds **22** (**TC490**) (0.033 g, 35% yield) and **22a** (**TC491**) (0.013 g, 14%) were synthesized from 2-(1-(3,5-dimethoxyphenethyl)-1*H*-1,2,3-triazol-4-yl)-4-(4-methoxybenzyl)-2,5,7,8-tetramethyl-3,4-dihydro-2*H*-benzo[*b*][1,4]oxazine (**20**) (0.11 g, 0.21 mmol) following the general procedure above.

**22** (**TC490**): ^1^H NMR (600 MHz, CD_3_OD): *δ* 7.37 (s, 1H), 6.43 (s, 1H), 6.16 (d, *J* = 2.4 Hz, 1H), 6.04 (d, *J* = 2.4 Hz, 2H), 4.50 (dt, *J* = 30.5, 6.9 Hz, 2H), 3.57 (d, *J* = 12.1 Hz, 1H), 3.31 (d, *J* = 12.1 Hz, 1H), 3.04–2.88 (m, 2H), 2.13 (s, 3H), 2.08 (s, 3H), 2.03 (s, 3H), 1.63 (s, 3H) (Figure S10); ^13^C NMR (151 MHz, CD_3_OD): *δ* 159.7, 152.1, 141.6, 140.8, 129.0, 127.3, 124.4, 123.7, 122.7, 122.0, 108.1, 102.1, 72.8, 52.5, 50.7, 37.6, 25.4, 19.4, 16.7, 11.5 (Figure S11); ESI-HRMS (*m*/*z*): calcd for C_22_H_27_N_4_O_3_ [M + H]^+^ 395.2078; found 395.2073 (Figure S12).

**22a** (**TC491**): ^1^H NMR (600 MHz, CD_3_OD): *δ* 7.39 (s, 1H), 6.77 (d, *J* = 8.6 Hz, 2H), 6.62 (d, *J* = 8.6 Hz, 2H), 6.13 (t, *J* = 2.2 Hz, 1H), 6.01 (d, *J* = 2.2 Hz, 2H), 4.57–4.43 (m, 2H), 3.89 (s, 2H), 3.56 (d, *J* = 12.2 Hz, 1H), 3.32 (d, *J* = 12.2 Hz, 1H), 3.06–2.85 (m, 2H), 2.13 (s, 3H), 2.05 (s, 3H), 1.94 (s, 3H), 1.62 (s, 3H) (Figure S13); ^13^C NMR (150 MHz, CD_3_OD): *δ* 159.8, 156.2, 152.2, 140.8, 140.5, 132.9, 130.5, 129.8, 129.0, 127.0, 123.8, 123.0, 121.8, 116.0, 108.2, 102.1, 72.6, 52.6, 51.0, 37.7, 35.3, 25.4, 15.8, 13.2, 12.2 (Figure S14); ESI-HRMS (*m*/*z*): calcd for C_29_H_33_N_4_O_4_ [M + H]^+^ 501.2496; found 501.2497 (Figure S15).

**5-(2-(4-(((4-(4-methoxybenzyl)-2,5,7,8-tetramethyl-3,4-dihydro-2H-benzo[*b*][1,4]oxazin-2-yl)methoxy)methyl)-1H-1,2,3-triazol-1-yl)ethyl)benzene-1,3-diol (26, TC484) and 3-methoxy-5-(2-(4-(((4-(4-methoxybenzyl)-2,5,7,8-tetramethyl-3,4-dihydro-2H-benzo[*b*][1,4]oxazin-2-yl)methoxy)methyl)-1H-1,2,3-triazol-1-yl)ethyl)phenol (26a)**

The title compounds (**26**, **TC484**) (0.021 g, 29% yield) and **26a** (traces) were synthesized from 2-(((1-(2-(2,2-dimethylbenzo[*d*][1,3]dioxol-5-yl)ethyl)-1H-1,2,3-triazol-4-yl)methoxy)methyl)-4-(4-methoxybenzyl)-2,5,7,8-tetramethyl-3,4-dihydro-2*H*-benzo[*b*][1,4]oxazine (**25**) (0.07 g, 0.12 mmol) following the general procedure above.

**26** (**TC484**): ^1^H NMR (600 MHz, acetone-*d*_6_): *δ* 8.21 (brs, 2H), 7.78 (s, 1H), 7.41 (d, *J* = 8.4 Hz, 2H), 6.92 (d, *J* = 8.7 Hz, 2H), 6.51 (s, 1H), 6.22 (dd, *J* = 11.8, 2.2 Hz, 3H), 4.64 (s, 2H), 4.57 (t, *J* = 7.6 Hz, 2H), 4.29 (d, *J* = 15.8 Hz, 1H), 4.19 (d, *J* = 15.8 Hz, 1H), 3.79 (s, 3H), 3.59 (d, *J* = 9.3 Hz, 1H), 3.43 (d, *J*= 9.3 Hz, 1H), 3.15 (d, *J*= 13.9 Hz, 1H), 3.04 (t, *J* = 7.6 Hz, 2H), 2.76 (d, *J* = 13.9 Hz, 1H), 2.16 (s, 3H), 2.13 (s, 3H), 2.04 (s, 3H), 1.29 (s, 3H) (Figure S16); ^13^C NMR (150 MHz, acetone-*d*_6_): *δ* 159.7, 159.6, 144.8, 144.7, 140.9, 132.4, 132.0, 129.8, 129.3, 127.1, 125.2, 124.2, 122.3, 114.6, 108.0, 102.0, 74.2, 73.3, 65.1, 58.1, 55.5, 51.7, 51.6, 37.2, 23.3, 19.5, 19.3, 11.9 (Figure S17); ESI-HRMS (*m*/*z*): calcd for C_32_H_39_N_4_O_5_ [M + H]^+^ 559.2915; found 559.2910 (Figure S18).

**26a**: ^1^H NMR (600 MHz, acetone-*d*_6_): *δ* 7.78 (s, 1H), 7.41 (d*, J* = 8.6 Hz, 2H), 6.92 (d, *J* = 8.6 Hz, 2H), 6.51 (s, 1H), 6.30 (t, *J* = 1.8 Hz, 1H), 6.27–6.23 (m, 2H), 4.63 (s, 2H), 4.60 (t, *J* = 7.5 Hz, 2H), 4.28 (d, *J*= 15.8 Hz, 1H), 4.18 (d, *J*= 15.8 Hz, 1H), 3.79 (s, 3H), 3.67 (s, 3H), 3.58 (d, *J*= 9.3 Hz, 1H), 3.42 (d, *J* = 9.3 Hz, 1H), 3.14 (d, *J* = 13.9 Hz, 1H), 3.09 (t, *J* = 7.5 Hz, 2H), 2.76 (d, *J* = 13.9 Hz, 1H), 2.16 (s, 3H), 2.13 (s, 3H), 2.03 (s, 3H), 1.29 (s, 3H); ^13^C NMR (150 MHz, acetone-*d*_6_): *δ* 162.0, 159.7, 159.6, 144.8, 144.7, 140.8, 132.4, 132.0, 129.8, 129.3, 127.1, 125.2, 124.2, 122.3, 114.6, 109.1, 106.4, 100.6, 74.2, 73.3, 65.1, 58.1, 55.5, 55.4, 51.7, 51.6, 37.3, 23.3, 19.5, 19.3, 11.9; ESI-HRMS (*m*/*z*): calcd for C_33_H_41_N_4_O_5_ [M + H]^+^ 573.3071; found 573.3067.

### 2.2. Cells and Culture Conditions

FF95 fibroblast cultures, previously established by outgrowth from foreskin of a one-year-old healthy donor [61], were kindly provided by Prof. Karin Scharffetter-Kochanek. Cells were routinely cultured in Dulbecco’s Modified Eagle Medium (DMEM; PAN Biotech GmbH, Aidenbach, Germany) supplemented with antibiotics, 100 IU/mL of penicillin and 100 μg/mL of streptomycin (Biosera, Nuaillé, France), as well as 10% (*v*/*v*) fetal bovine serum (FBS; Life Technologies Europe BV, Thessaloniki, Greece), in a humidified environment of 5% CO_2_ and 37 °C. Cells were routinely subcultured once a week at a 1:2 split ratio, using a trypsin (Life Technologies Europe BV)–citrate (0.25%–0.3% *w*/*v*) solution. Cell counting following trypsinization and suspension in IsoFlow Sheath Fluid (Beckman Coulter Diagnostics, Brea, CA, USA) was performed using a Coulter counter (Beckman Coulter Diagnostics). Cells were tested for mycoplasma contamination at regular intervals and found to be mycoplasma-free.

For obtaining senescent fibroblast cultures, FF95 cells were exposed to sublethal repeated doses of ionizing radiation, as previously reported [62]. Briefly, cells received a cumulative dose of 50 Gy through their exposure to a ^60^Co gamma source (Gamma Chamber 4000 A, Isotope Group, Bhadha Atomic Research Company, Trombay, Bombay, India) at a rate of 2.5 Gy/min. The cultures were then left to grow and passaged (2–3 times) until they exhibited less than 5% 5-bromo-2′-deoxyuridine (BrdU) incorporation and more than 90% senescence-associated β-galactosidase (SA-β-Gal) staining, hence considered to be senescent [63]. In particular for SA-β-Gal staining, cells were sparsely plated on glass coverslips, left to grow for 5 days, then fixed with 3% (*v*/*v*) formaldehyde in PBS. Staining was implemented through incubation with 40 mM citric acid/sodium phosphate (pH 6.0) supplemented with 150 mM NaCl, 2 mM MgCl_2_, 5 mM potassium ferricyanide, 5 mM potassium ferrocyanide, and 1 mg/mL X-Gal at 37^0^C. SA-β-Gal-positive or -negative cells were observed under an ECLIPSE Ts2 inverted microscope (Nikon, Tokyo, Japan) and photographs were captured through a Basler acA1920-155ucMIC camera (Basler AG, Ahrensburg, Germany).

### 2.3. Radical Scavenging Assay

Radical scavenging capacity of the test compounds was assessed by the 2,2-Diphenyl-1-Picrylhydrazyl (DPPH) method, as described previously [64]. Briefly, appropriate compound dilutions in dimethyl sulfoxide (DMSO; Sigma, St. Louis, MO, USA) were mixed with equal volumes of a freshly prepared DPPH (Sigma) solution (1 mM), and the absorbance at 520 nm was monitored at regular time intervals using a FLUOstar Optima microplate reader (BMG Labtech, Ortenberg, Germany). DMSO was used as negative control, while the water-soluble vitamin E analogue 6-hydroxy-2,5,7,8-tetramethylchroman-2-carboxylic acid (Trolox) served as positive control.

### 2.4. Cytotoxicity Assay

Cytotoxicity was estimated by a modification of the 3-(4,5-Dimethylthiazol-2-yl)-2,5-diphenyltetrazolium bromide (MTT) assay [65]. Briefly, FF95 fibroblasts were plated in 96-well flat-bottomed transparent microplates in DMEM 10% FBS at a density of 10,000 cells/well. After an overnight incubation to ensure cell attachment, serial dilutions of the test compounds in culture medium were added and left to act on the cells for 72 h. Subsequently, the medium was replaced by serum-free, phenol-red-free DMEM (PAN Biotech GmbH) containing 1 mg/mL of MTT (Sigma), incubated for four hours; the MTT-formazan crystals formed were solubilized in 2-propanol (Sigma) and the absorbance was measured using a Spark multimode microplate reader (Tecan Group Ltd., Männedorf, Switzerland) at a wavelength of 550 nm (reference wavelength 690 nm). Corresponding vehicle (DMSO) dilutions were used as control.

### 2.5. Intracellular Reactive Oxygen Species (ROS) Levels

FF95 fibroblasts were plated, in clear-bottomed black 96-well microplates in DMEM 10% FBS at a density corresponding to the 1:2 split ratio. When the cultures have reached confluency, the medium was replaced by a serum-free one, containing non-cytotoxic concentrations of the test compounds. Following an overnight incubation, 2′,7′-dichlorodihydrofluorescein diacetate (DCFH-DA; Sigma) was added at a final concentration of 10 μM, and—at various time-points—fluorescence emission was monitored at 520 nm following excitation at 485 nm, using a FLUOstar Optima microplate reader. DMSO-treated cell cultures served as negative control and Trolox was used as a positive control at a final concentration of 20 μΜ. Beyond basal ROS levels estimation, in order to assess the capability of the test compounds to prevent intracellular ROS level-stimulation, 1.5 mM H_2_O_2_ in serum free medium was added after the 30 min incubation with DCFH-DA, and the fluorescence emission was measured as above [64].

### 2.6. Cellular Glutathions (GSH) Levels

An adaptation of the microplate assay based on the fluorescent probe monochlorobimane (mCB) was used for the assessment of the glutathione (GSH) levels in human skin fibroblasts [66]. Briefly, FF95 fibroblasts were plated in clear-bottomed black 96-well microplates in DMEM 10% FBS at a density corresponding to the 1:2 split ratio. When the cultures reached confluency, the medium was replaced by the phenol-red-free, serum-free one, containing non-cytotoxic concentrations of the test compounds, and the cells were incubated for 20 h. At the end of the incubation period, the medium was replaced with 5 μM mCB (MedChemExpress, New Jersey, NJ, USA) diluted in Hanks’ Balanced Salt Solution (HBSS) for a further 4 h, and fluorescence was recorded in a Spark (Tecan) plate reader using an excitation wavelength of 380 nm and emission wavelength of 480 nm. Treatment of the cells with 8 mM N-ethylmaleimide (NEM; Sigma), previously reported to deplete cellular GSH (Hedley et al., 1994), was used to assess nonspecific background labelling. N-acetycysteine (NAC; Sigma) at 10 mM was used as positive control [67], and vehicle (DMSO) as negative control.

### 2.7. RNA Extraction and Gene Expression Analysis

Gene expression analysis was based on quantitative real-time polymerase chain reaction (qRT-PCR) [63]. Total RNA was extracted using Trizol (Life Technologies Europe BV), following the manufacturer’s instructions, from FF95 cell cultures growing in DMEM 10% FBS in the presence or absence of non-cytotoxic concentrations of the test compounds for 24 h. First-strand complementary DNA (cDNA) was synthesized from 500 ng of the total RNA using PrimeScript™ RT Reagent Kit according to the manufacturer’s instructions (Takara Bio Inc, Tokyo, Japan). Five microliters of the cDNA (1:25) per sample was subjected to qRT-PCR using the KAPA SYBR FAST qPCR kit (KAPA Biosystems, Wilmington, MA, USA). The reaction was carried out in an MX 3000 P QPCR Systems Cycler (Stratagene, La Jolla, CA, USA), and data analysis was performed with MxPro version 4.1 QPCR software (Stratagene, La Jolla, CA, USA). Relative mRNA levels compared to untreated cells were estimated as described before [63] with glyceraldehyde-3-phosphate dehydrogenase (GAPDH) serving as the reference gene. The ΔΔCt method was used to evaluate the relative messenger RNA expression of each gene. The primers used for amplification were *ho-1*: forward 5′-GCC CTT CAG CAT CCT CAG TTC C-3′, reverse 5′- AGT GGT CAT GGC CGT GTC AAC-3′; *gapdh*: forward 5′-GAG TCC ACT GGG GTC TTC-3′, reverse 5′-GCA TTG CTG ATG ATC TTG GG-3′.

## 3. Results

### 3.1. Chemical Synthesis

**Scheme 1:**
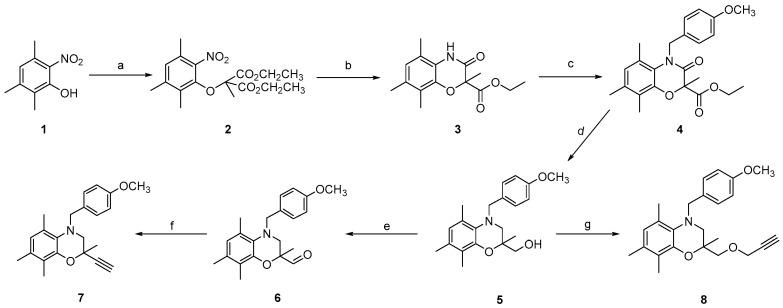
Synthesis of alkynes **7**, **8**. Reagents and conditions: (**a**) Diethyl-2-bromo-2-methyl malonate, Cs_2_CO_3_, TBAI, DMF, 40 °C, (**b**) Na_2_S_2_O_4_, EtOH:H_2_O (4:5), 60 °C, (**c**) *p*-methoxybenzylchloride, K_2_CO_3_, TBAB, 80 °C, (**d**) (CH_3_)_2_SBH_3_, THF, 67 °C, (**e**) Cl_2_CHCOOH, N,N’-diisopropylcarbodiimide, DMSO, rt, (**f**) Bestmann–Ohira reagent, K_2_CO_3_, CH_3_OH, 0 °C → rt, (**g**) Propargyl bromide, NaH, DMF, 0 °C → rt.

**Scheme 2:**
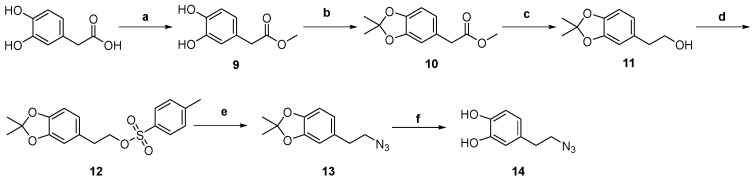
Synthesis of azides **13, 14**. Reagents and conditions: (**a**) CH_3_OH, H_2_SO_4_, 65 °C, (**b**) (CH_3_)_2_C(OCH_3_)_2_, camphor-10- sulfonic acid, CHCl_3_, 65 °C, (**c**) LiAlH_4_, THF, 0 °C → rt, (**d**) TsCl, Et_3_N, CH_2_Cl_2_, 0 °C → rt, (**e**) NaN_3_, DMF, rt, (**f**) TFA:H_2_O (5:1), CHCl_3_, 0 °C → rt.

**Scheme 3:**
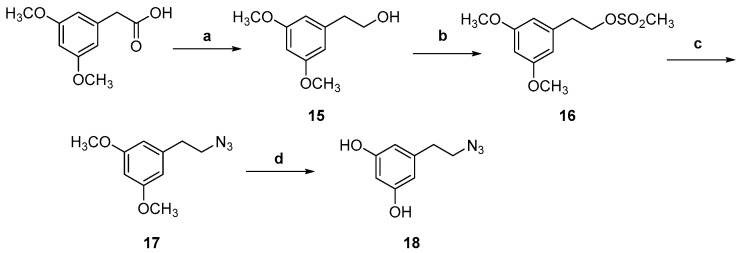
Synthesis of azides **17, 18**. Reagents and conditions: (**a**) LiAlH_4_, THF, 0 °C → rt, (**b**) CH_3_SO_2_Cl, Et_3_N, CH_2_Cl_2_, 0 °C → rt, (**c**) NaN_3_, DMF, rt, (**d**) BF_3_S(CH_3_)_2_, CH_2_Cl_2_, 0 °C → rt.

**Scheme 4:**
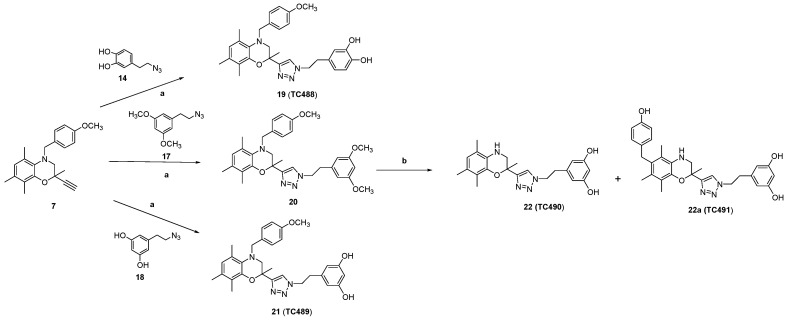
Synthesis of final compounds **19** and **21**, **22**, **22a**. Reagents and conditions: (**a**) CH_2_Cl_2_:H_2_O (1:1), CuSO_4_.5H_2_O/NaAsc, rt, (**b**) BF_3_S(Me)_2_, DCM, 0 °C → rt.

**Scheme 5:**
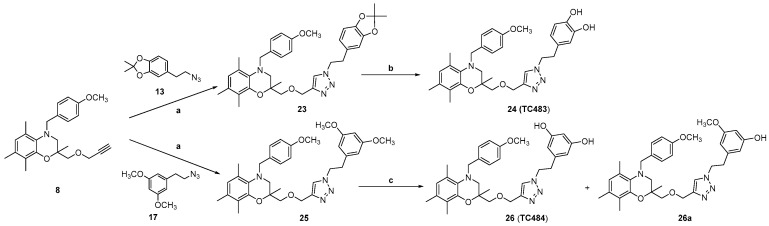
Synthesis of final compounds **24** (**TC483**) and **26** (**TC484**). Reagents and conditions: (**a**) *t*BuOH:H_2_O (1:1), CuSO_4_∙5H_2_O/NaAsc, MW(90 °C/80W), **(b**) TFA:H_2_O (5:1), CHCl_3_, 0 °C → rt, (**c**) BF_3_S(CH_3_)_2_, CH_2_Cl_2_, 0 °C → rt.

The synthesis of 5,7,8-trimethyl-1,4-benzoxazine analogues **19**, **21**, **22**, **22a**, **24**, **26**, and **26a** bearing at position 2 a triazole ring is depicted in Scheme 1, Scheme 2, Scheme 3, Scheme 4 and Scheme 5. In particular, the synthesis of the 1,2,3-triazole-substituted benzoxazines **19**, **20**, **21**, **23**, and **25** was effected through a click reaction between alkyne **7** or **8** and azides **13**, **14**, **17**, and **18**. The required alkynes **7** and **8** were prepared as described in Scheme 1. Initially the required 5,7,8-trimethyl-benzoxazinone **3** was synthesized according to a previously reported method of our group [41] with modified reductive conditions [68]. Thus, alkylation of 2,3,5-trimethyl-6-nitrophenol (**1**) with 2-bromo-2-methylmalonic acid diethyl ester afforded analogue **2**, which upon reduction using Na_2_S_2_O_4_ in EtOH:H_2_O (4:5 *v*/*v*) at 60 °C and spontaneous cyclization gave benzoxazinone **3**. Then, *N*-alkylation using *p*-methoxybenzyl chloride in the presence of K_2_CO_3_ and a catalytic amount of TBAB gave compound **4**, which was reduced by (CH_3_)_2_SBH_3_ in THF at 67 °C to the corresponding alcohol **5**. Oxidation of **5** to the aldehyde **6** under Pfitzner–Moffatt conditions [69], followed by reaction with dimethyl (diazomethyl)phosponate (Bestmann–Ohira reagent) [70,71,72], generated in situ from dimethyl-1-diazo-2-oxopropylphosphonate by treatment with K_2_CO_3_ in CH_3_OH) led to the desired alkyne **7**. Finally, alkyne **8** was obtained by alkylation of alcohol **5** with propargyl bromide in DMF using NaH as a base (Scheme 1).

Azides **13**, **14** and **17**, **18** were synthesized from the commercially available (3,4-dihydroxyphenyl)acetic acid and (3,5-dimethoxylphenyl)acetic acid, respectively as described in Scheme 2 and Scheme 3. Briefly, esterification of (3,4-dihydroxyphenyl)acetic acid with methanol, in the presence of a catalytic amount of H_2_SO_4_ to the methyl ester **9** was followed by protection of the catechol moiety as the acetonide **10**. Reduction of the ester in compound **10** with LiAlH_4_ to alcohol **11** followed by formation of the corresponding tosylate **12** [73] and reaction with NaN_3_ in DMF gave azide **13**. TFA-mediated removal of the acetonide protecting group afforded the final azide **14** (Scheme 2). Reduction of (3,5-dimethoxylphenyl)acetic acid with LiAlH_4_ gave the corresponding alcohol **15**, which was transformed to the respective mesylate ester **16** [74]. Reaction of **16** with NaN_3_ in DMF gave the corresponding azide **17 [74]** in high yield (90%). Deprotection of the methoxy groups to obtain the desired analogue **18** was achieved by using BF_3_·S(CH_3_)_2_ complex in CH_2_Cl_2_ [73] (Scheme 3).

Following the synthesis of the desired alkynes (**7**, **8**) and azides (**13**, **14, 17**, and **18**) 1,2,3-triazole-substituted benzoxazines were prepared regioselectively through a Cu^1^-catalyzed ‘click’ cycloaddition reaction [75,76]. The click reaction was carried out in the presence of CuSO_4_·5H_2_O and sodium ascorbate (NaAsc) in a mixture of CH_2_Cl_2_/H_2_O (1:1) under conventional conditions or *t*BuOH/H_2_O (1:1) under microwave-assisted irradiation, as shown in Scheme 4 and Scheme 5. Thus, the catecholic final product **19** (**TC488**) was obtained by the cycloaddition reaction of alkyne **7** and azide **14** at room temperature in 57% yield. In turn, click reaction between alkyne **7** and the protected azide **17** at room temperature afforded the 1,2,3-triazolyl derivative **20** in 67% yield. Deprotection of the methoxy groups in **20** using BF_3_S(Me)_2_ also resulted in the removal of the *p*-methoxybenzyl group affording compound **22** (**TC490**) in 35% yield. In addition, we obtained the byproduct derivative **22a** (**TC491**) in 14% yield resulting from the migration of the *p*-hydroxybenzyl group from *N*-4 to C6 of the 5,7,8-trimethyl-1,4-benzoxazine system. Thus, in order to obtain the deprotected resorcinol derivative **21** (**TC489**) bearing the *p*-methoxybenzyl group at *N*-4 for structure activity studies, we successfully performed the cycloaddition reaction with alkyne **7** and the deprotected azide **18** and **TC489** were isolated in 67% yield (Scheme 4).

The synthesis of derivatives bearing a methoxymethyl-1,2,3-triazolyl spacer **24** (**TC483**) and **26** (**TC484**) were prepared from the click reaction of alkyne **8** and azide **13** or **17** to afford derivatives **23** and **25**, respectively. Deprotection of the acetonide group in **23** using TFA afforded derivative **24** (**TC483**) in 81% yield. In turn, deprotection of the methoxy groups in **25** using BF_3_S(CH_3_)_2_ afforded the resorcinol deprotected derivative **26** (**TC484**) in 29% yield accompanied by traces of compound **26a** in which only one methoxy group of the resorcinol moiety was deprotected (Scheme 5).

The structural characterization of all the synthesized compounds was accomplished by ^1^H and ^13^C NMR spectroscopy and (high resolution) mass spectrometry.

### 3.2. Free-Radical Scavenging Activity of the Test Compounds

**Figure 2:**
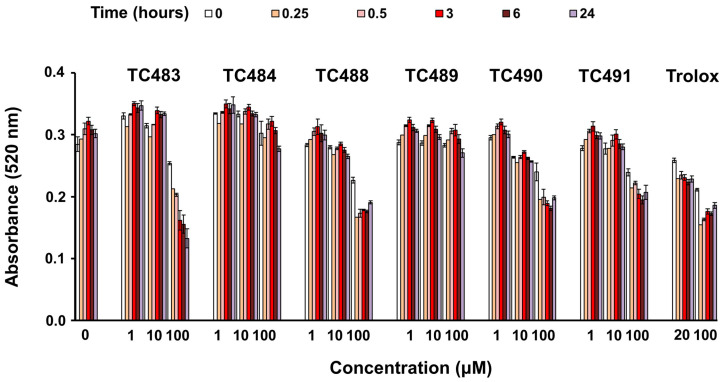
DPPH-scavenging activity of the test compounds. The indicated concentrations of the test compounds were co-incubated with DPPH for the indicated time-intervals, and the absorbance was measured as described under Materials and Methods. Average of two independent experiments (error-bars represent standard deviation).

The six final compounds **TC483**, **TC484**, **TC488**, **TC489**, **TC490**, and **TC491** were initially tested for their antioxidant capacity in a cell-free system, i.e., using the method of the free radical DPPH. As shown in Figure 2, four out of the six compounds were able to scavenge DPPH in a time- and concentration-dependent manner. The most active compounds seemed to be **TC483** and **TC488**, which exhibited scavenging activity similar to the positive control (Trolox, 100 μM) at the highest concentration tested (100 μM). **TC490** and **TC491** were also effective DPPH scavengers, especially at 100 μM. The remaining two compounds, i.e., **TC484** and **TC489** were marginally active at 100 μM and only following incubation for 24 and 3 h, respectively.

### 3.3. Cytotoxic Activity of the Test Compounds

**Figure 3:**
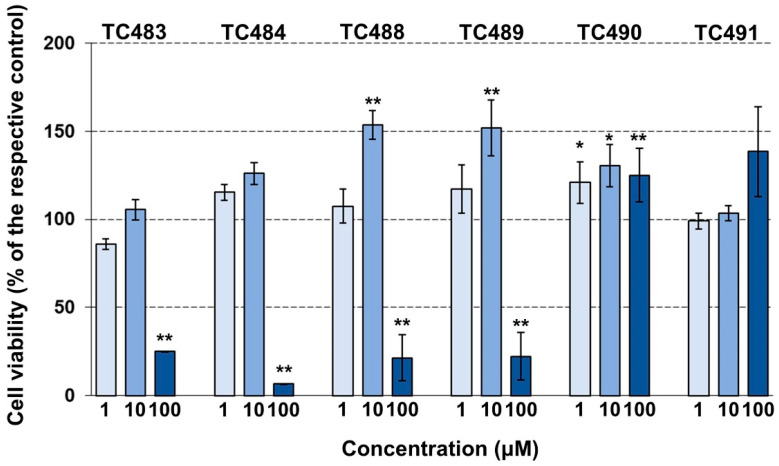
Cytotoxicity of the test compounds. Human skin fibroblasts were exposed to the indicated concentrations of the test compounds for 72 h, and their viability was assessed with the MTT method, as described under Materials and Methods. Average of three independent experiments; error-bars represent standard deviation (* *p* < 0.05; ** *p* < 0.01).

Then, the effect of the six compounds on the viability of human skin fibroblasts was assessed based on the widely accepted MTT method. As shown in Figure 3, all the compounds were not cytotoxic at 1 or 10 μM concentration. However, **TC483**, **TC484**, **TC488**, and **TC489** were cytotoxic at the highest concentration tested, i.e., 100 μM. On the other hand, **TC488** and **TC489** increased cellular viability at 10 μM. However, testing viability with an alternative method (see Supplementary Materials, Figure S19) indicated no difference from the vehicle. **TC490** was not cytotoxic at any concentration tested, and furthermore it mildly increased cellular viability. Finally, **TC491** did not exhibit any statistically significant effect at any concentration tested.

### 3.4. Intracellular Reactive Oxygen Species (ROS) Levels

**Figure 4:**
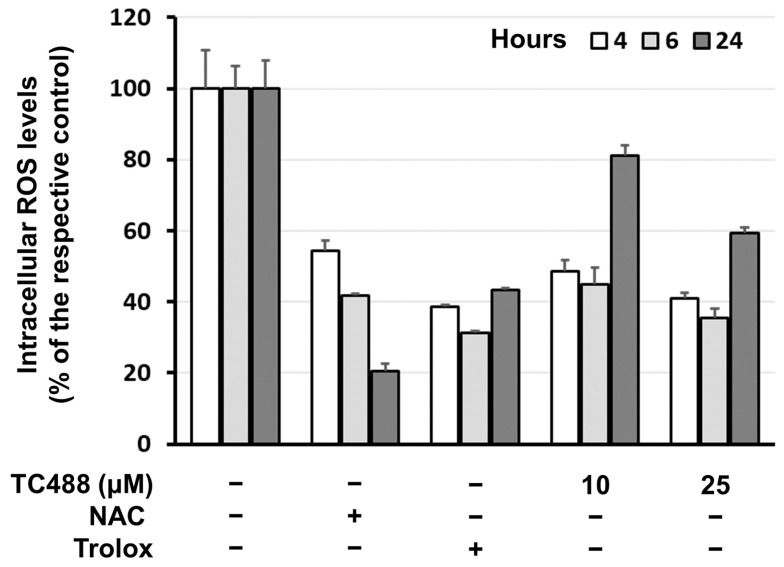
Intracellular ROS levels of human skin fibroblasts in the presence of **TC488**. Human skin fibroblasts were treated overnight with the indicated concentrations of **TC488** or 5 mM NAC or 20 μM Trolox in serum-free medium, then DCFH-DA was added and fluorescence was monitored at the indicated time-intervals, as described under Materials and Methods. Average of three independent experiments; error-bars represent standard deviation.

Based on the above results, **TC488** was considered as the most promising, since it was one of the most active radical scavengers (Figure 2) and at 10 μM it stimulated cell viability (Figure 3). Hence, it was further evaluated for its intracellular antioxidant activity by means of the DCFH-DA probe, which is internalized by the cells and upon oxidation by the cells’ reactive oxygen species (ROS) yields the highly fluorescent DCF. As shown in Figure 4, **TC488** at both 10 μM and 25 μM and at early time-points (up to 6 h) suppressed the intracellular ROS levels of human skin fibroblasts at a level comparable to that of the potent antioxidants NAC and Trolox. At later time-points, ROS levels tend to recover to control levels, especially in the presence of 10 μM **TC488**.

**Figure 5:**
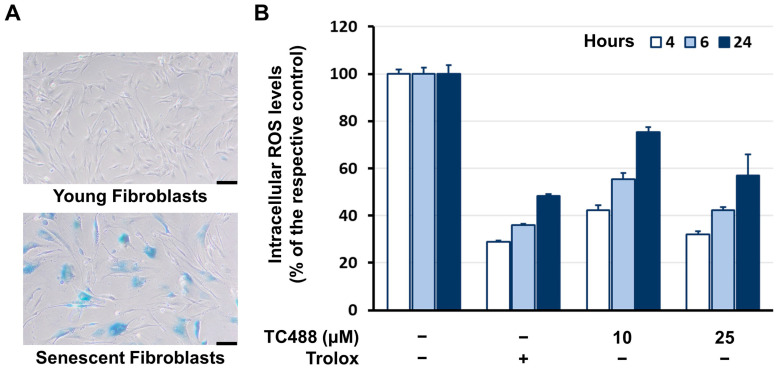
Intracellular ROS levels of senescent human skin fibroblasts in the presence of **TC488**. (**A**) Representative photographs of human skin fibroblasts rendered senescent (as described under Materials and Methods) vs. their young counterparts, following their staining with SA-β-Gal (magnification 10×; scale bar=100 μm). (**B**) Senescent human skin fibroblasts were treated overnight with the indicated concentrations of **TC488** or 20 μM Trolox in serum-free medium, then DCFH-DA was added and fluorescence was monitored at the indicated time-intervals, as described under Materials and Methods. Average of three independent experiments; error-bars represent standard deviation.

Interestingly, **TC488** had similar effects on human skin fibroblasts that have been rendered senescent through repeated mild doses of ionizing radiation (stress-induced premature senescence; SIPS). In Figure 5A, the validation of cell senescence through SA-β-Gal staining is depicted. As shown in Figure 5B, despite the higher ROS levels that are characterizing senescent cells, **TC488** had a dose-dependent antioxidant effect, interestingly comparable to those of the positive control Trolox.

**Figure 6:**
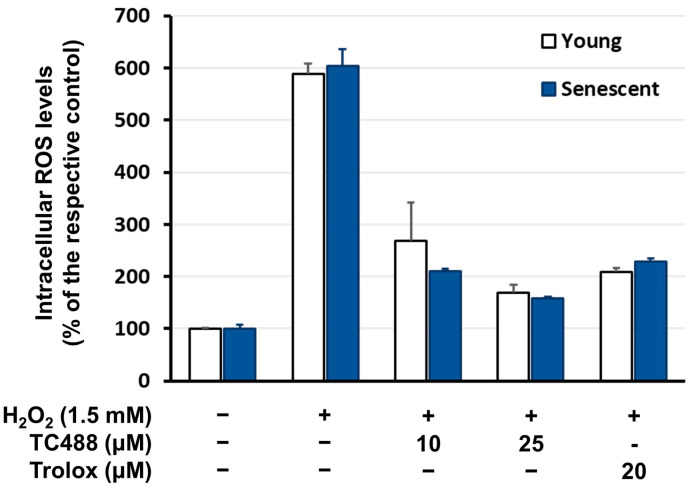
Reversal of oxidative-stress-induced ROS levels by **TC488** in young and senescent human skin fibroblasts. Human skin fibroblasts were pre-treated for 3 h with the indicated concentrations of **TC488** or 20 μM Trolox in serum-free medium, then DCFH-DA along with 1.5 mM H_2_O_2_ was added and fluorescence was monitored after 1 h, as described under Materials and Methods. Average of two independent experiments; error-bars represent standard deviation.

Beyond the suppression of ROS basal levels, **TC488** was also capable of reverting hydrogen peroxide induction of ROS in both young and senescent human skin fibroblasts (Figure 6).

**Figure 7:**
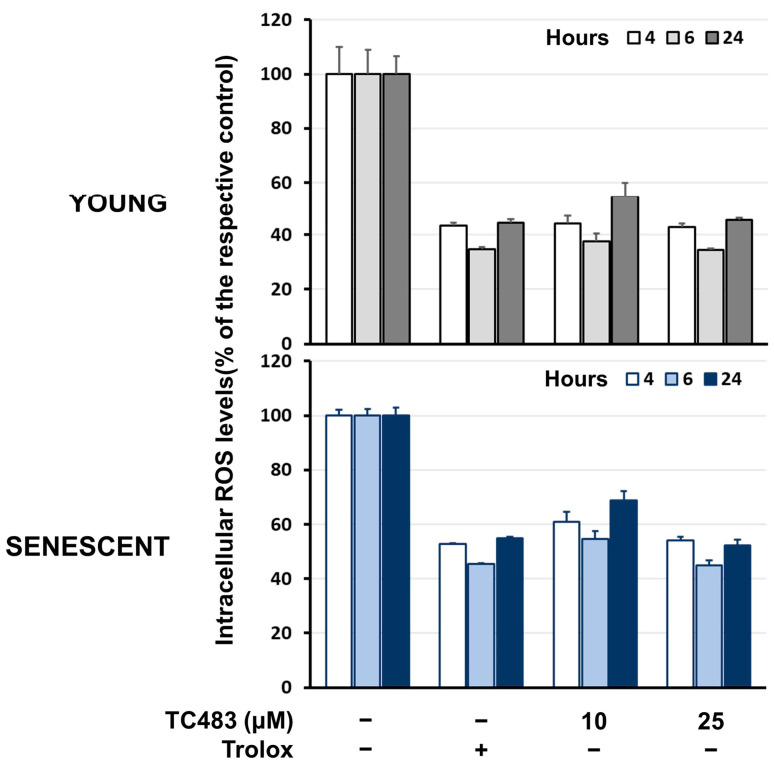
Intracellular ROS levels of young and senescent human skin fibroblasts in the presence of **TC483**. Cells were treated overnight with the indicated concentrations of **TC483** or 20 μM Trolox in serum-free medium, then DCFH-DA was added and fluorescence was monitored at the indicated time-intervals, as described under Materials and Methods. Average of three independent experiments; error-bars represent standard deviation.

**Figure 8:**
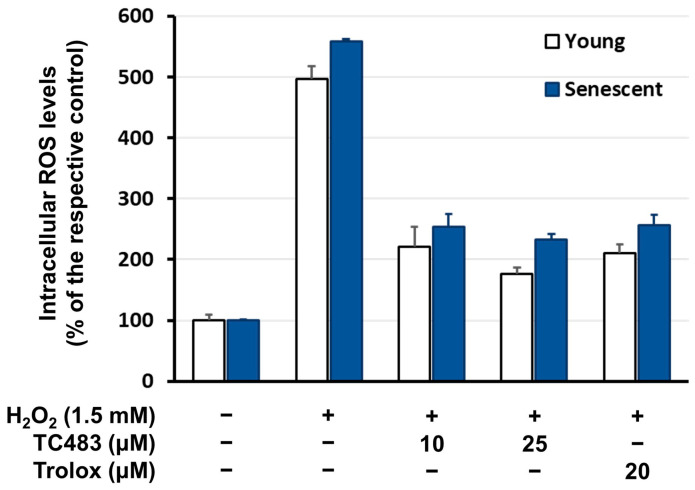
Reversal of oxidative-stress-induced ROS levels by **TC483** in young and senescent human skin fibroblasts. Human skin fibroblasts were pre-treated for 3 h with the indicated concentrations of **TC483** or 20 μM Trolox in serum-free medium, then DCFH-DA along with 1.5 mM H_2_O_2_ was added and fluorescence was monitored after 1 h, as described under Materials and Methods. Average of two independent experiments; error-bars represent standard deviation.

Besides **TC488**, analogue **TC483** was also studied for its effects on the intracellular ROS levels of both young and senescent human skin fibroblasts, and it was found to suppress them at a level comparable to that of the positive control Trolox, with a slight tendency for recovery at 24 h (Figure 7). **TC483** (like **TC488**) was also capable of reverting H_2_O_2_-induction of ROS in both young and senescent human skin fibroblasts (Figure 8).

**Figure 9:**
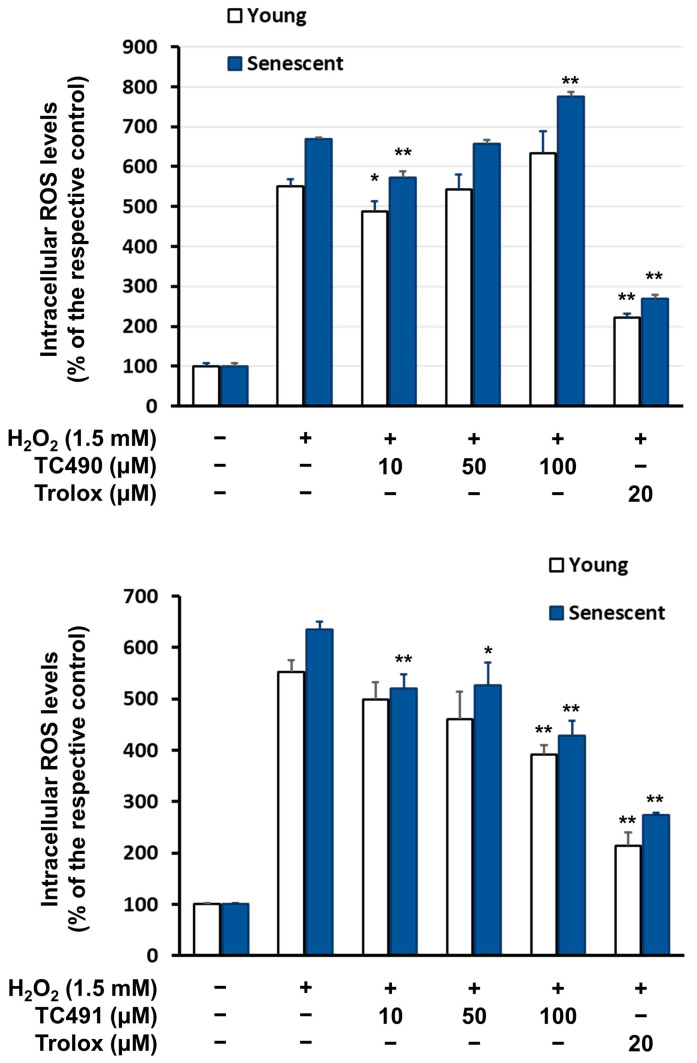
Reversal of oxidative-stress-induced ROS levels by **TC490** and **TC491** in young and senescent human skin fibroblasts. Human skin fibroblasts were pre-treated for 3 h with the indicated concentrations of the two compounds or 20 μM Trolox in serum-free medium, then DCFH-DA along with 1.5 mM H_2_O_2_ was added and fluorescence was monitored after 1 h, as described under Materials and Methods. Average of two independent experiments; error-bars represent standard deviation; asterisks denote statistically significant differences in comparison to control cells (Student’s *t*-test, * *p*< 0.05, ** *p* < 0.01).

Compounds **TC490** and **TC491**, although less effective than **TC483** and **TC488** as free radical scavengers (Figure 2) were also tested for their effect on intracellular ROS levels at a broader concentration range (since they were not cytotoxic at any concentration tested, see Figure 3). They were not effective in inhibiting basal ROS levels; however, **TC490** at 10 μM was capable of a mild—yet statistically significant—inhibition of H_2_O_2_-stimulated ROS in both young and senescent skin fibroblasts, while it increased ROS levels at 100 μM (Figure 9, upper panel). Moreover, **TC491** exhibited a dose-dependent mild inhibition of H_2_O_2_-stimulated oxidative stress, especially in the case of senescent cells, where all tested concentrations were active, while in young cells only the highest concentration had a statistically significant antioxidant effect (Figure 9, lower panel).

### 3.5. Effect of the Test Compounds on Gene Expression

**Figure 10:**
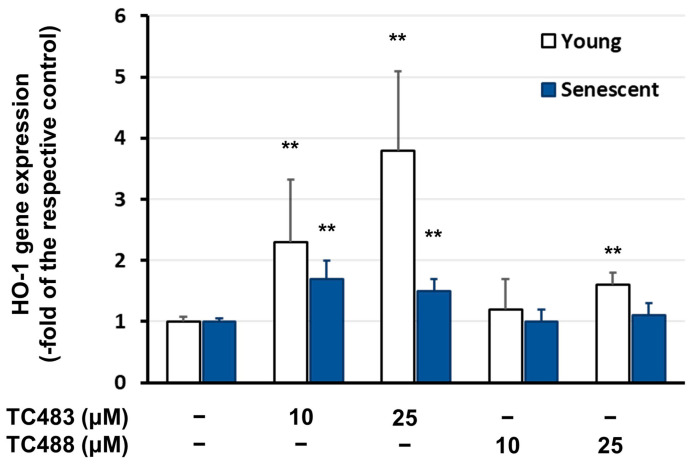
HO-1 gene expression in young and senescent human skin fibroblasts following treatment with **TC483** and **TC488**. Human skin fibroblasts were treated for 24 h with the indicated concentrations of the two compounds, then total mRNA was collected and gene expression studied, as described under Materials and Methods. Average of three independent experiments; error-bars represent standard deviation (** *p* < 0.01).

Since **TC483** and **TC488** were found to attenuate ROS levels, we studied their effect on the expression of *heme oxygenase-1* (*ho-1*), a known target of the transcription factor Nrf2, the latter regulating the cellular antioxidant defense systems [77,78,79]. As shown in Figure 10, a 24 h incubation with both 10 μΜ and 25 μM **TC483** led to statistically significant induction of *ho-1* gene expression in both young and senescent human skin fibroblasts. The induction was higher in young than in senescent cells.

**TC488** on the other hand, was not effective at the concentration of 10 μΜ, while at 25 μM induced *ho-1* gene expression only in young fibroblasts (Figure 10).

### 3.6. Effect of the Test Compounds on Glutathione (GSH) Levels

**Figure 11:**
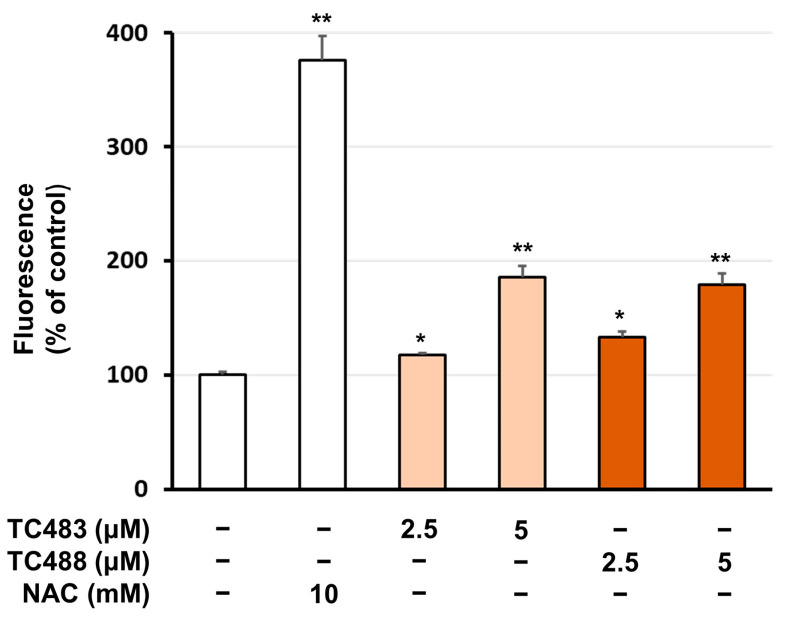
GSH levels in human skin fibroblasts following treatment with **TC483** and **TC488**. Human skin fibroblasts were treated overnight with the indicated concentrations of the two compounds or 10 mM NAC, then GSH levels were assessed using 5 μM mCB, as described under Materials and Methods. Average of two independent experiments; error-bars represent standard deviation (* *p* < 0.05; ** *p* < 0.01).

The effects of **TC483** and **TC488** on cellular GSH levels were assessed by using the probe mCB. As shown in Figure 11, an overnight incubation with both compounds led to a dose-dependent increase of GSH levels. Hence, these two promising compounds seem to confer further antioxidant protection to human skin fibroblasts through GSH induction.

## 4. Discussion

In the current work we report the synthesis and evaluation of the antioxidant activity of six novel hybrid compounds bearing the 5,7,8-trimethyl-1,4-benzoxazine core and a catechol (**TC483** and **TC488**), or resorcinol group (**TC484**, **TC489**, **TC490**, and **TC491**) connected through a heteroaromatic linker, namely a 1,2,3-triazole. In compounds **TC488, TC489, TC490**, and **TC491** the triazole ring is directly attached at C2 of the benzoxazine moiety, while in **TC483** and **TC484** the attachment is through a methoxymethyl spacer. Compounds **TC483** and **TC488** substituted by the ethyl-3,4-dihydroxyphenyl group can be envisioned as hybrids with hydroxytyrosol, the latter being known for its broad-spectrum biological properties.

Concerning the cell-free antioxidant capacity of the hybrids the catechol bearing analogues, **TC483** and **TC488** exhibited the highest scavenging activity, similar to Trolox. Conversely, the resorcinol compounds **TC484** and **TC489** were marginally active following incubation for 24 and 3 h, respectively. Concerning the effect of the hybrids on the viability of human skin fibroblasts, the methoxybenzyl analogues **TC483**, **TC484**, **TC488**, and **TC489** were cytotoxic at the highest concentration of 100 μM. In contrast, the catechol compound **TC488** and its resorcinol congener **TC489** at 10 μM enhanced human skin fibroblast viability by approx. 50% over the control, as judged by the MTT assay, but they did not show any effect based on the alternative Neutral Red assay (Figure S19). This may indicate an effect of the compounds on the mitochondrial enzymes responsible for MTT reduction without true effect on viability or a direct interaction between the compounds and MTT [80,81]. Regarding the resorcinol analogues obtained following the removal of the methoxy benzyl group, **TC490** exhibited a different activity profile, mildly supporting cell viability at all concentrations tested, while **TC491** did not exhibit any statistically significant effect at any concentration. Interestingly, in **TC491**, the C6 of the benzoxazine moiety bears a 4-hydroxybenzyl group which suggests that a free C6 position plays an important role in enhancing cell viability. The most promising hybrid from the cell-free antioxidant and cell viability tests (**TC488**) was found to suppress reactive oxygen species (ROS) at both 10 μM and 25 μM and at early time-points (up to 6 h) in both young and senescent human skin fibroblasts, at a level comparable to that of the positive controls NAC and Trolox. The methoxymethyl spacer-bearing analogue of **TC488**, compound **TC483**, was also capable of reducing intracellular ROS levels in both young and senescent human skin fibroblasts, at a level comparable to that of the positive control Trolox, with a slight tendency for recovery at 24 h. Among the less active derivatives **TC490** and **TC491**, **TC490** at 10 μM was active against intracellular ROS in both young and senescent skin fibroblasts, while **TC491** was potent mainly in the case of senescent cells. These results show that deprotection of the 1,4-benzoxazine nitrogen is not beneficial for activity. The intracellular antioxidant activity of **TC483** and **TC488** was further confirmed by their ability to increase GSH levels in a dose-dependent manner. Since Nrf2 is the main transcription factor that regulates the expression of the cellular antioxidant defense system, **TC483** and **TC488** were assessed for their action on Nrf2’s downstream effector, *ho-1*. **TC483** resulted to an induction of ho-1 gene expression in both young and senescent human skin fibroblasts (at 10 μM and 25 μM) with preference for young cells, while **TC488** led to statistically significant induction of the *ho-1* gene expression in young human skin fibroblasts only at 25 μM. It should be noted here that other redox-sensitive transcription factors, such as activator protein-1 (AP-1), nuclear factor-kappa B (NF-kB), and hypoxia inducible factor (HIF), may also regulate *ho-1*; however, Nrf2 is being considered as its main transcriptional regulator [82,83].

Generally, the synthesized compounds have been shown to possess antioxidant activity for human skin fibroblast cultures, while no quantitative differences were observed between the response of young and senescent cells. Of course, a variety of stimuli, such as oxidative stress, ionizing radiation, ultraviolet radiation, etc., may lead to senescent cells with different characteristics. Hence, a different behavior of cells rendered senescent through other methods than the one used in the present study cannot be ruled-out. The current study is limited to cell-free and in vitro assay systems. The results presented here are promising; however, in vivo studies may be necessary to assess the safety and tolerance profile of the synthesized compounds prior to their further applications.

## 5. Conclusions

In conclusion, six novel 5,7,8-trimethyl-1,4-benzoxazine/catechol or resorcinol hybrids were synthesized connected through a methoxymethyl-1,2,3-triazolyl or a 1,2,3-triazoly linker. The compounds were evaluated for their antioxidant activity in cell-free system as well as in young and senescent human skin fibroblasts. Overall, the catechol derivatives **TC483** and **TC488** were more potent than the resorcinol analogues **TC484**, **TC489**, **TC490**, and **TC491**. Compounds **TC483** and **TC488**, were shown to act coordinately as radical scavengers, ROS inhibitors, *ho-1* gene expression inducers, and GSH enhancers. All these properties make these compounds putative useful ingredients for usage in anti-ageing applications including the cosmetic industry, especially in products targeting skin ageing. The effect of these compounds on the expression of other features of senescent cells is currently under investigation.

## Data Availability

All data support for this research is included in this article and Supplementary File.

## Supplementary Materials

The following supporting information can be downloaded at: https://www.mdpi.com/article/10.3390/antiox13070798/s1, Experimental procedures for compounds **2**, **3**, and **9**–**18**. ^1^H and ^13^C NMR and HR-MS spectra of the final compounds Figures S1–S18. Experimental procedure for the Neutral Red assay and Figure S19. Cytotoxicity of the test compounds at 10 μM, as assessed with the Neutral Red method.
