# Supplementary material for: Design and Synthesis of Novel Antioxidant 2-Substituted-5,7,8-Trimethyl-1,4-Benzoxazine Hybrids: Effects on Young and Senescent Fibroblasts
Source: Antioxidants (Basel). 2024 Jun 29;13(7):798. doi: 10.3390/antiox13070798 (PMC11274006; doi:10.3390/antiox13070798)
Supplement: Supplementary file 1 [file antioxidants-13-00798-s001.zip › antioxidants-3057582-supplementary.pdf]

Table of Contents

1.Experimental Procedures for the synthesis of compounds 2-3 and 9-18......S2

2.Copies of ^{1}H-NMR, ^{13}C-NMR and HRMS spectra of final compounds........S6

3.Experimental Procedure for Neutral Red Assay S18

1. Experimental Procedures for the synthesis of compounds 2-3 and 9-18

1.1. Diethyl 2-methyl-2-(2,3,5-trimethyl-6-nitrophenoxy)malonate (2)

To a solution of 2-nitro-3,5,6-trimethylphenol (**1**) (2.29 g, 12.64 mmol) in dry DMF (32.5 ml) was added Cs_2CO_3 (8.92 g, 25.28 mmol), TBAI (catalytic amount), and 2-bromo-2-methyl-propionic acid ethyl ester (4.84 ml, 25.28 mmol) and the mixture was heated at 40 °C overnight. Subsequently, the reaction mixture was diluted with water and extracted with ethyl acetate. The organic layer was washed with brine and dried over Na_2SO_4. The solvent was evaporated *in vacuo*, and the crude product was purified by flash column chromatography (petroleum ether 40-60 °C/diethyl ether 90:10 v/v) to afford compound **2** as a colorless oil (4.33 g, 97% yield). ^{1}H NMR (600 MHz, $CDCl_3$): 6.88 (s, 1H), 4.31 (q, J = 7.2 Hz, 4H), 2.25 (s, 3H), 2.20 (s, 3H), 2.12 (s, 3H), 1.53 (s, 3H), 1.33 (t, J = 7.2 Hz, 6H); ^{13}C NMR (150 MHz, $CDCl_3$): δ 168.6, 146.3, 143.7, 140.1, 131.6, 128.8, 127.3, 85.0, 62.7, 20.5, 18.2, 16.8, 14.1, 13.4; ESI-MS (m/z): 353.83 $[M+H]^+$, 375.98 $[M+Na]^+$.

1.2. Ethyl 2,5,7,8-tetramethyl-3-oxo-3,4-dihydro-2H-benzo[b][1,4]oxazine-2-carboxylate (3)

To a solution of diethyl 2-methyl-2-(2,3,5-trimethyl-6-nitrophenoxy)malonate (**2**) (4.16 g, 11.77 mmol) in 78 ml EtOH:H_2O (4:5), $Na_2S_2O_4$ (30 g, 17.20 mmol) was added and the reaction mixture was stirred at 60 °C overnight. Upon completion, the solvent was concentrated under reduced pressure and the residue was diluted with H_2O and extracted with EtOAc. The organic layer was washed with brine, dried over Na_2SO_4, filtered and the solvent was evaporated *in vacuo*. The residue was triturated with ethanol and then with petroleum ether to afford compound **3** as a white solid (3.11 g, 95% yield). ^{1}H NMR (300 MHz, $CDCl_3$): δ 7.87 (brs, 1H), 6.63 (s, 1H), 4.21–4.03 (m, 2H), 2.20 (s, 6H), 2.17 (s, 3H), 1.86 (s, 3H), 1.14 (t, J = 7.2 Hz, 3H); ^{13}C NMR (75 MHz, $CDCl_3$): δ 169.0, 164.8, 141.3, 132.6, 125.7, 123.6, 122.3, 120.3, 80.8, 62.2, 20.7, 19.5, 16.0, 14.0, 11.6; ESI-MS (m/z): 278.01 $[M+H]^+$.

1.3. Methyl 2-(3,4-dihydroxyphenyl)acetate (9)

To a solution of 2-(3,4-dihydroxyphenyl)acetic acid (1.71 g, 10.2 mmol) in methanol (140 mL) conc. H_2SO_4 was added (10 drops). The reaction mixture was then refluxed, in the dark, for 2 h. Upon completion of the reaction, the mixture was cooled to ambient temperature and evaporated *in vacuo* almost to dryness. The residue was diluted with ethyl acetate and washed with sat. aq. $NaHCO_3$ solution. The organic layer was washed with brine, dried over anhydrous Na_2SO_4 and evaporated to dryness. Methyl 2-(3,4-dihydroxyphenyl)acetate (**9**) was obtained as a dark yellow oil which was used to the next step without further purification. ^{1}H NMR (300 MHz, $CDCl_3$): δ 6.82 – 6.70 (m, 2H), 6.65 (dd, J = 8.1, 2.0 Hz, 2H), 3.71 (s, 3H), 3.52 (s, 2H); ^{13}C NMR (75 MHz, $CDCl_3$): δ 173.7, 143.9, 143.2, 126.2, 121.9, 116.4, 115.6, 52.5, 40.6; ESI-MS (m/z): 181.43 $[M-H]^-$.

1.4. Methyl 2-(2,2-dimethylbenzo[d][1,3]dioxol-5-yl)acetate (10)

To a solution of methyl 2-(3,4-dihydroxyphenyl)acetate (**9**) (1.56 g, 8.57 mmol) in dry CH_2Cl_2 (85 mL), were added 2,2-dimethoxypropane (10.5 mL, 85.7 mmol) and camphorsulfonic acid (0.38 g, 1.63 mmol). The reaction mixture was refluxed in the dark for 24 h. Upon completion of the reaction, the mixture was cooled to ambient temperature and sat. aq. $NaHCO_3$ solution was added to pH 7. The mixture was then diluted with ethyl acetate and the organic layer was washed with brine, dried over anhydrous Na_2SO_4 and evaporated to dryness. Compound **10** was obtained after FCC (petroleum ether/EtOAc 95:5 v/v) as a colorless oil (1.39 g, 73% yield). ^{1}H NMR (600 MHz, $CDCl_3$): *δ* 6.74–6.61 (m, 3H), 3.69 (s, 3H), 3.51 (s, 2H), 1.66 (s, 6H); ^{13}C NMR (75 MHz, $CDCl_3$): δ 172.3, 147.7, 146.6, 126.9, 121.8, 118.0, 109.6, 108.2, 52.1, 40.9, 25; ESI-MS (m/z): 245.00 $[M+Na]^+$, 466.60 $[2M+Na]^+$.

1.5. 2-(2,2-Dimethylbenzo[d][1,3]dioxol-5-yl)ethan-1-ol (11)

To an ice-cold suspension of $LiAlH_4$ (0.26 g, 6.95 mmol,) in dry THF (10 mL), was added a solution of methyl 2-(2,2-dimethylbenzo[d][1,3]dioxol-5-yl)acetate (**10**) (1.03 g, 4.63 mmol) in dry THF (16 mL) dropwise. The reaction mixture was stirred at ambient temperature for 1 h. Upon completion of the reaction, the excess amount of $LiAlH_4$ was quenched by the addition of a solution THF/H_2O 95:5 dropwise at 0 °C. The reaction mixture was then diluted with ethyl acetate and anhydrous Na_2SO_4 was added. The mixture was filtered through a celite pad and the filtrate was evaporated *in vacuo*. The title compound **11** was afforded as a yellow oil and used in the next step without further purification. ^{1}H NMR (600 MHz, $CDCl_3$): *δ* 6.74–6.55 (m, 3H), 3.81 (t, *J* = 6.5 Hz, 2H), 2.77 (t, *J* = 6.4 Hz, 2H), 1.67 (s, 6H); ^{13}C NMR (150 MHz, $CDCl_3$): *δ* 147.8, 146.2, 131.5, 121.4, 117.9, 109.2, 108.3, 63.9, 39.0, 26.0; ESI-MS (m/z): 245.00 $[M+Na]^+$, 466.60 $[2M+Na]^+$.

1.6. 2-(2,2-Dimethylbenzo[d][1,3]dioxol-5-yl)ethyl 4-methylbenzenesulfonate (12)

To an ice-cold solution of 2-(2,2-dimethylbenzo[d][1,3]dioxol-5-yl)ethan-1-ol (**11**) (0.88 g, 4.53 mmol) in dry CH_2Cl_2 (19 mL), Et_3N (5.1 ml, 38.2 mmol) was added dropwise. Then, a solution of *p*-toluenesulfonyl chloride (2.15 g, 11.3 mmol) in dry CH_2Cl_2 (10 mL) was added dropwise at 0 °C and the reaction mixture was stirred at ambient temperature for 19 h. Upon completion of the reaction, the mixture was diluted with CH_2Cl_2, washed with brine, dried over anhydrous Na_2SO_4 and evaporated to dryness. Compound **12** was obtained as a colorless crystalline solid (1.49 g, 89% yield) after purification by FCC (petroleum ether/EtOAc 85:15 v/v). ^{1}H NMR (600 MHz, $CDCl_3$): *δ* 7.72 (d, *J* = 8.2 Hz, 2H), 7.30 (d, *J* = 8.0 Hz, 2H), 6.59 (d, *J* = 7.8 Hz, 1H), 6.51 – 6.49 (m, 2H), 4.14 (t, *J* = 7.2 Hz, 2H), 2.84 (t, *J* = 7.2 Hz, 2H), 2.44 (s, 3H), 1.65 (s, 6H); ^{13}C NMR (75 MHz, $CDCl_3$): *δ* 147.6, 146.4, 144.7, 133.1, 129.8, 129.2, 127.9, 121.4, 117.9, 109.1, 108.2, 70.9, 35.2, 25.9, 21.7; ESI-MS (m/z): 370.76 $[M+Na]^+$.

1.7. 2-(3,5-dimethoxyphenyl)ethan-1-ol (15)

To an ice-cold suspension of $LiAlH_4$ (0.19 g, 5.10 mmol) in 3 mL dry THF, a solution of 2-(3,5-dimethoxyphenyl)acetic acid (0.50 g, 2.55 mmol) in 2.5 mL dry THF was added. The reaction mixture was gradually warmed to room temperature and then was stirred at the same temperature for 2.5 h. Then, it was quenched by dropwise addition

of water, diluted with ethyl acetate and filtered. Extraction with ethyl acetate. The organic layer was washed with saturated aqueous NH_4Cl, brine and dried over Na_2SO_4. Solvent was evaporated *in vacuo*. Compound **15** was obtained as a yellow oil, (0.450 g, 97% yield) and was used to the next step without further purification. ^{13}C NMR (150 MHz, $CDCl_3$): δ 161.0, 141.0, 107.1, 98.5, 63.5, 55.4, 39.6. ESI-MS (m/z): 182.93 $[M+H]^+$, 204.93 $[M+Na]^+$, 386.66 $[2M+Na]^+$.

1.8. 3,5-Dimethoxyphenethyl methanesulfonate (16)

To a solution of 2-(3,5-dimethoxyphenyl)ethan-1-ol (**15**) (0.41 g, 2.24 mmol) in 15 mL dry CH_2Cl_2, Et_3N (0.8 mL) and CH_3SO_2Cl (0.3 mL, 4.48 mmol) were added at 0 °C and the reaction mixture was stirred at room temperature for 2 h. After completion of the reaction, water was added and then extracted with CH_2Cl_2. The organic layer was washed with saturated aqueous $NaHCO_3$, NaCl, dried over Na_2SO_4, filtered and the solvent was evaporated *in vacuo*. Compound **16** was used without further purification as yellow oil (0.57 g, 98% yield). ^1H NMR (300 MHz, $CDCl_3$): δ 6.43 – 6.28 (m, 2H), 4.41 (t, J = 6.9 Hz, 2H), 3.78 (s, 6H), 2.99 (t, J = 7.0 Hz, 2H), 2.89 (s, 3H); ^{13}C NMR (75 MHz, $CDCl_3$): δ 161.1, 138.7, 107.1, 99.0, 70.2, 55.4, 55.4, 37.5, 36.0; ESI-MS (m/z): 260.84 $[M+H]^+$, 282.87 $[M+Na]^+$.

1.9. General procedure for the synthesis of azides 13, 17

To a solution of sulfonate **12** or **16** (1 eq) in dry DMF (0.3 M), NaN_3 (2 eq) was added and the mixture was stirred at 45 °C for 24 h. Upon completion of the reaction (checked by TLC), water was added and the mixture was extracted with diethyl ether. The organic layer was washed with saturated aqueous NaCl, dried over Na_2SO_4, filtered and the solvent was evaporated *in vacuo*. The desired product was obtained after FCC (petroleum ether/EtOAc 90:10 v/v).

1.9.1. 5-(2-Azidoethyl)-2,2-dimethylbenzo[d][1,3]dioxole (13)

Compound **13** was obtained following the above general procedure as a colorless oil (0.48 g, 90% yield) using 2-(2,2-dimethylbenzo[*d*][1,3]dioxol-5-yl)ethyl 4-methylbenzenesulfonate (**12**) (0.85 g, 2.44 mmol). ^1H NMR (600 MHz, $CDCl_3$): δ 6.67 - 6.60 (m, 3H), 3.45 (t, J = 7.2 Hz, 2H) 2.79 (t, J = 7.2 Hz, 2H), 1.66 (s, 6H); ^{13}C NMR (150 MHz, $CDCl_3$): δ 147.7, 146.4, 131.1, 121.2, 117.9, 108.97, 108.3, 52.8, 35.2, 25.9; ESI-MS (m/z): 220.11$[M+H]^+$, 242.09 $[M+Na]^+$.

1.9.2. 1-(2-Azidoethyl)-3,5-dimethoxybenzene (17)

Compound **17** was obtained following the above general procedure as colorless oil (0.65 g, 83%) using 3,5-dimethoxyphenethyl methanesulfonate (**16**) (0.98 g, 3.76 mmol). ^1H NMR (600 MHz, $CDCl_3$): δ 6.38 (d, J = 2.2 Hz, 2H), 6.36 (t, J = 2.2 Hz, 1H), 3.79 (s, 6H), 3.50 (t, J = 7.3 Hz, 2H), 2.83 (t, J = 7.3 Hz, 2H); ^{13}C NMR (150 MHz, $CDCl_3$): δ 161.1, 140.5, 107.0, 98.8, 55.4, 52.4, 35.8; APCI-HRMS (m/z): calcd for $C_{10}H_{14}O_5N_3$ $[M+H]^+$ 208.1081; found 208.1082.

1.10. 4-(2-Azidoethyl)benzene-1,2-diol (**14**)

To an ice-cold solution of 5-(2-azidoethyl)-2,2-dimethylbenzo[*d*][1,3]dioxole (**13**) (0.05 g, 0.23 mmol) in degassed $CHCl_3$ (6 ml), TFA (1.3 ml) and H_2O (0.26 ml) were added and the reaction was warmed at ambient temperature and stirred for 1h. Upon completion of the reaction (checked by TLC), the solvent was concentrated *in vacuo* and the residue was extracted with EtOAc. The organic layer was washed with saturated aqueous $NaHCO_3$, NaCl, dried over anhydrous Na_2SO_4, filtered, the solvent was evaporated to dryness under reduced pressure. The title compound **14** was obtained as a white solid (0.022 g, 81% yield) after FCC purification (hexanes/acetone 70:30 v/v). ^{1}H NMR (300 MHz, $CDCl_3$): δ 6.89 – 6.61 (m, 3H), 3.46 (t, J = 7.3 Hz, 2H), 2.78 (t, J = 7.2 Hz, 2H); ESI-MS (m/z): 177.94 $[M-H]^-$.

1.11. 5-(2-azidoethyl)benzene-1,3-diol (**18**)

To an ice-cold solution of 1-(2-azidoethyl)-3,5-dimethoxybenzene (**17**) (0.10 g, 0.48 mmol) in 4 ml dry CH_2Cl_2, $BF_3S(Me)_2$ (0.50 ml, 4.80 mmol) was added dropwise and the reaction mixture was stirred at ambient temperature for 24 h. After completion of the reaction, the solvent and excess reagent were evaporated under argon stream. The residue was extracted with ethyl acetate. The organic layer was washed with saturated aqueous $NaHCO_3$, NaCl, dried over Na_2SO_4 and the solvent was evaporated in vacuo. 5-(2-Azidoethyl)benzene-1,3-diol (**18**) was afforded after purification by FCC ($CHCl_3$/CH_3OH 93:7 v/v). ^{1}H NMR (600 MHz, CD_3OD): δ 6.19 (d, J = 2.1 Hz, 2H), 6.14 (t, J = 2.1 Hz, 1H), 3.44 (t, J = 7.1 Hz, 2H), 2.71 (t, J = 7.1 Hz, 2H); ESI-MS (m/z): 178.01 $[M-H]^-$.

2. Copies of ^{1}H-NMR, ^{13}C-NMR and HRMS spectra of final compounds

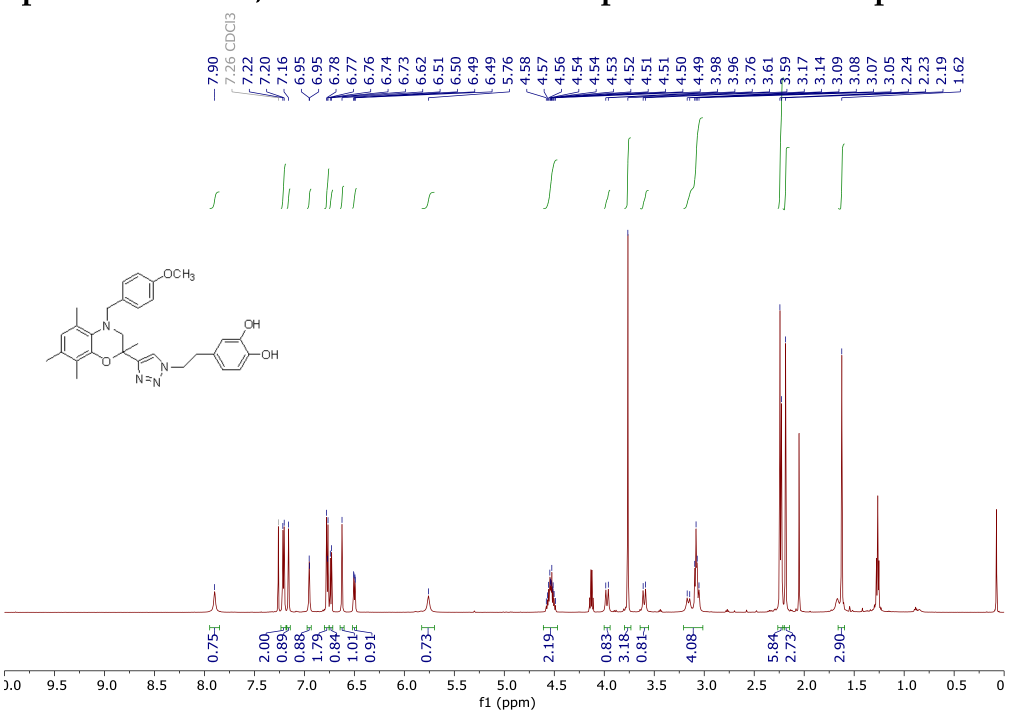

Figure S1. ^{1}H-NMR of compound **19 (TC488)**.

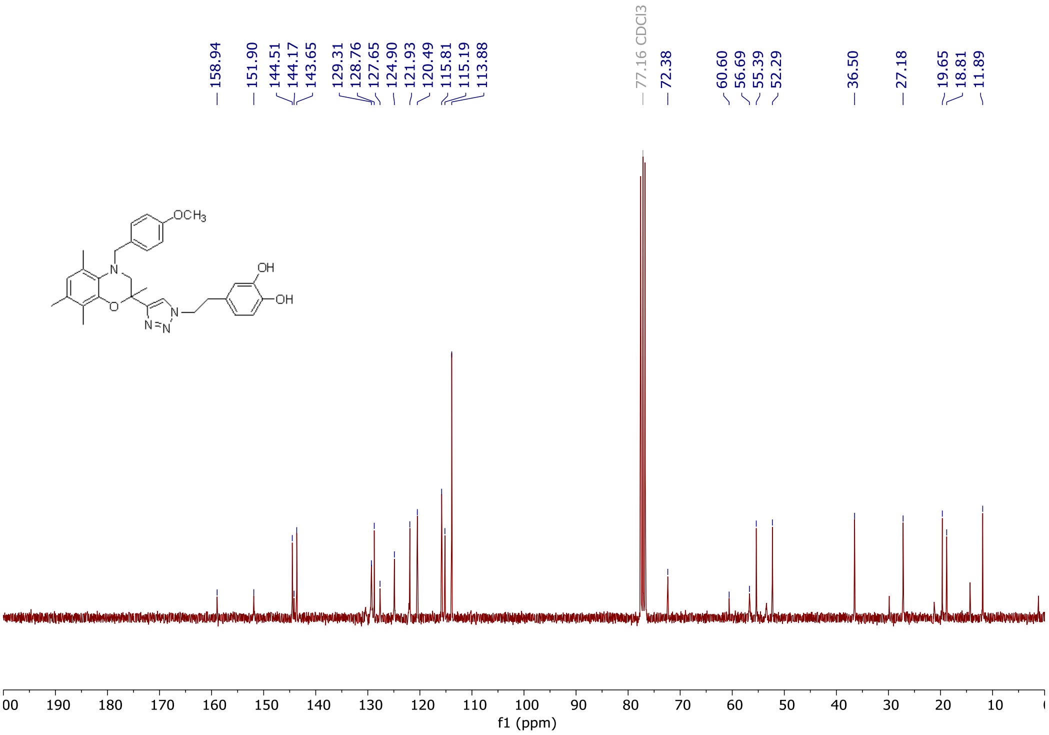

Figure S2. ^{13}C-NMR of compound **19 (TC488)**.

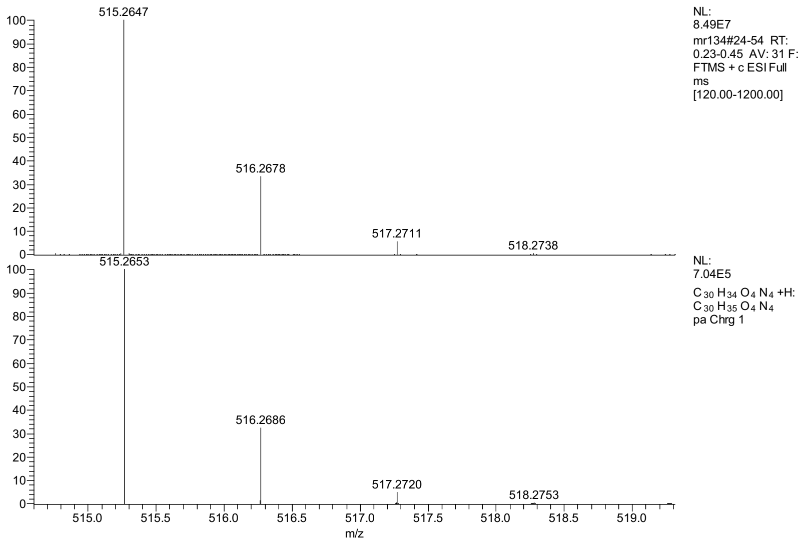

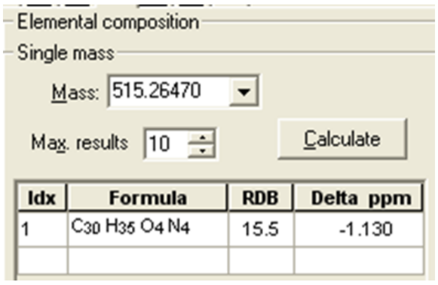
Elemental composition

Single mass

Mass: 515.26470

Max. results 10

Calculate

Idx	Formula	RDB	Delta ppm
1	$C_{30}H_{35}O_4N_4$	15.5	-1.130

Figure S3. HRMS of compound **19 (TC488)**.

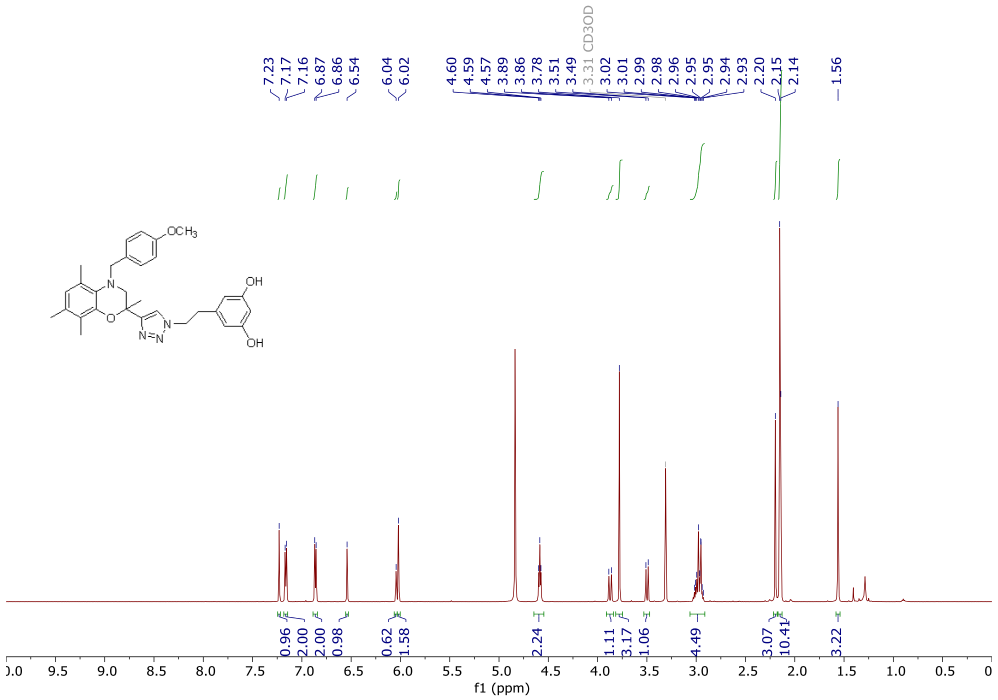

Figure S4. ^{1}H-NMR of compound **21 (TC489)**.

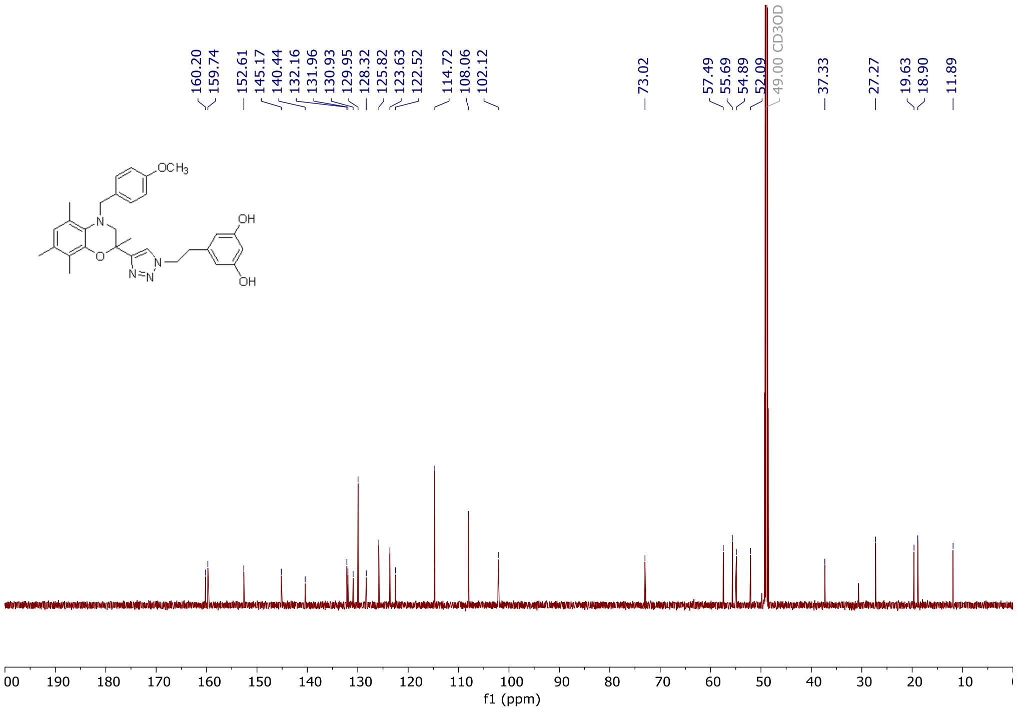

Figure S5. ^{13}C-NMR of compound **21 (TC489)**.

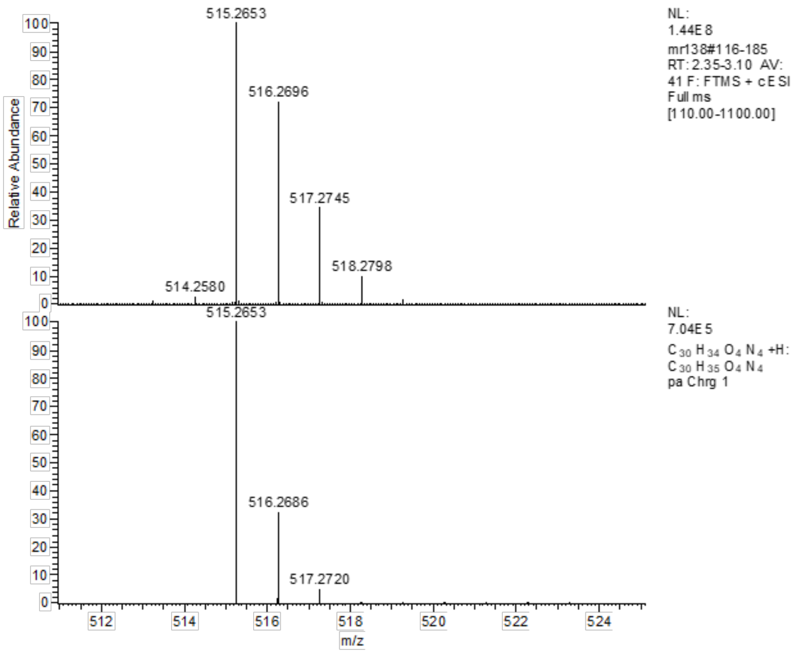

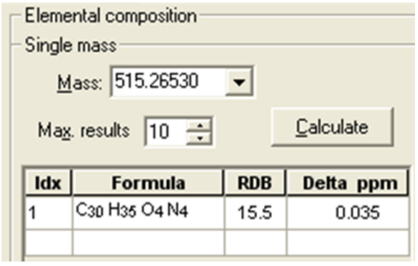

Figure S6. HRMS of compound **21 (TC489)**.

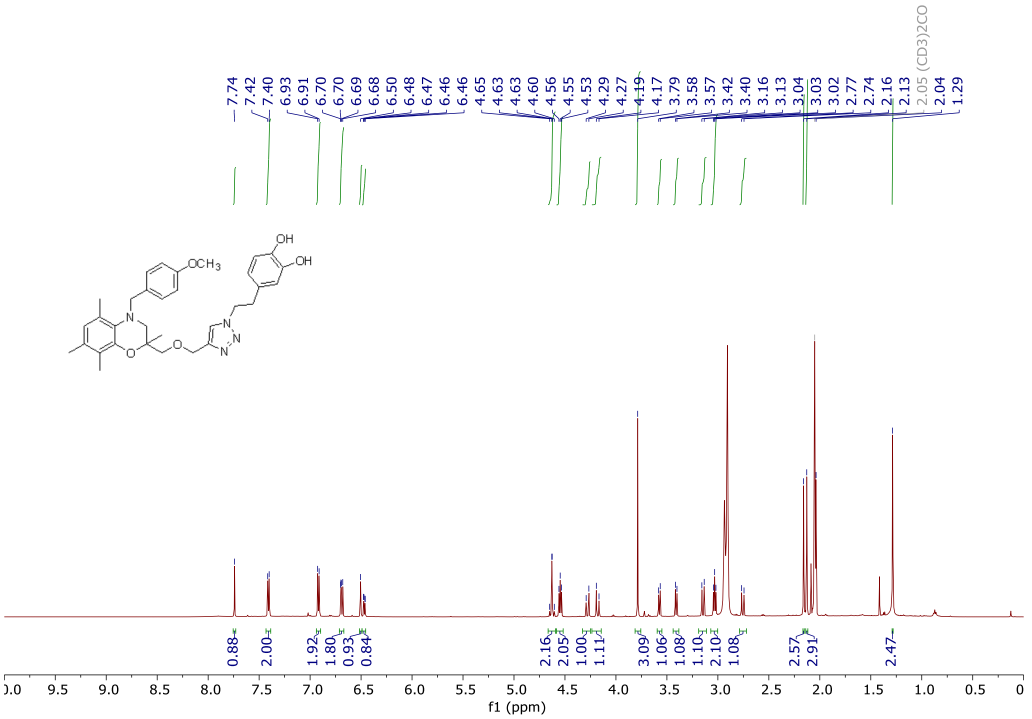

Figure S7. ^{1}H-NMR of compound **24 (TC483)**.

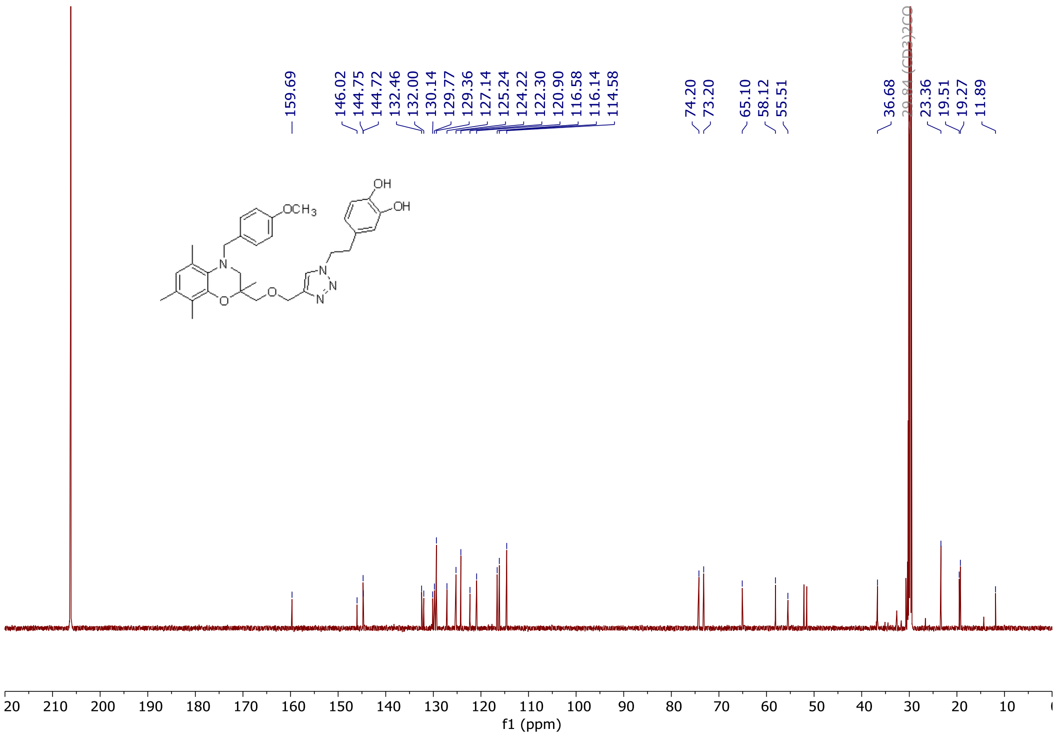

Figure S8. ^{13}C-NMR of compound **24 (TC483)**.

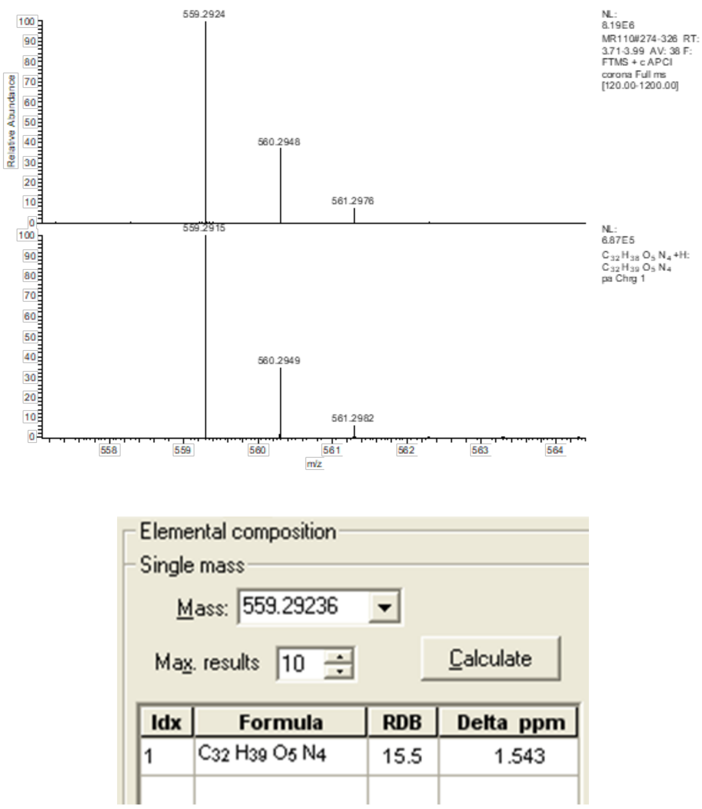

Figure S9. HRMS of compound **24 (TC483)**.

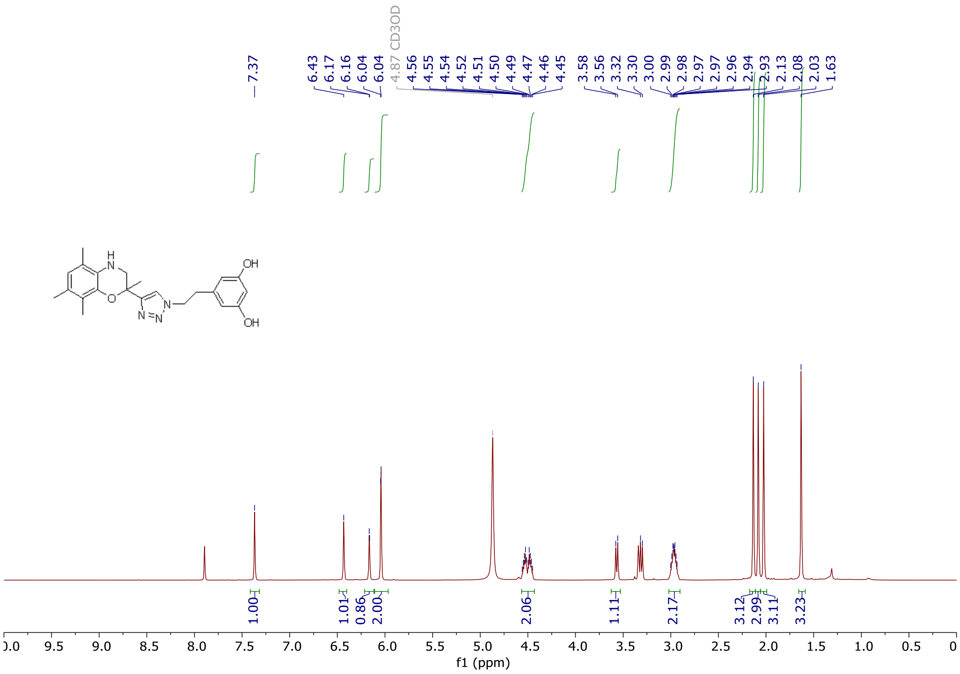

Figure S10. ^{1}H-NMR of compound **22 (TC490)**.

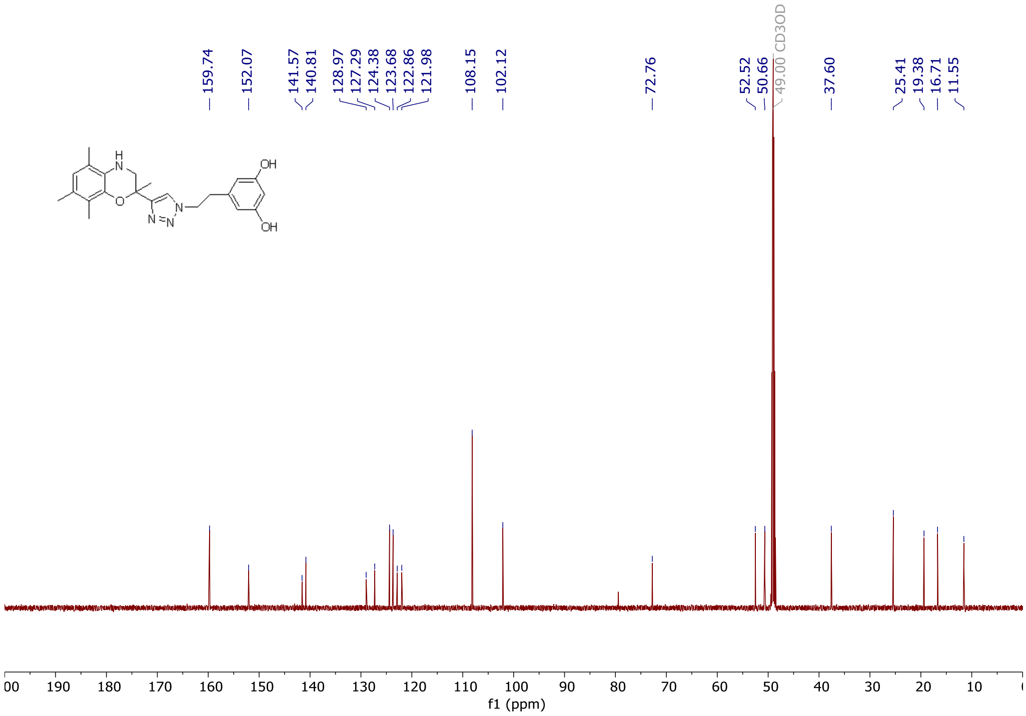

Figure S11. ^{13}C-NMR of compound **22 (TC490)**.

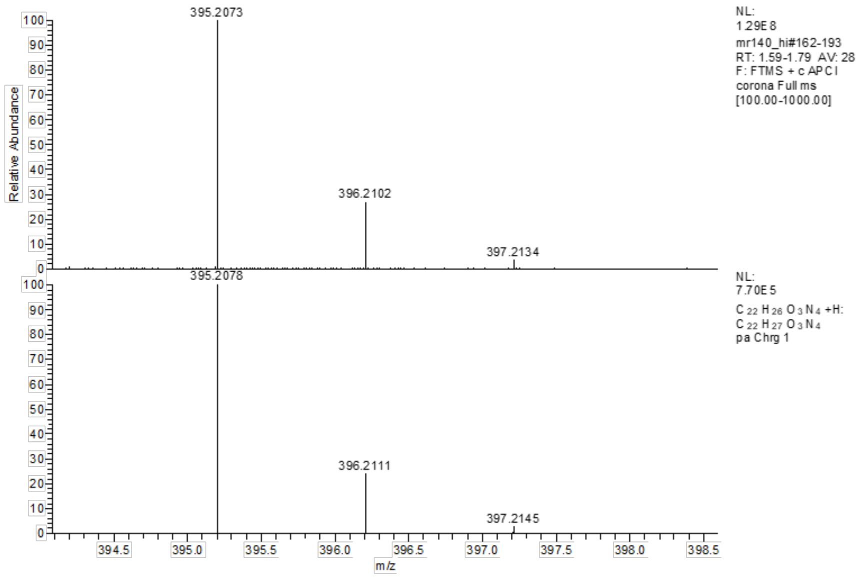

Elemental composition

Single mass

Mass: 395.20730

Max. results 10

Calculate

Idx	Formula	RDB	Delta ppm
1	$C_{22}H_{27}O_3N_4$	11.5	-1.182

Figure S12. HRMS of compound **22 (TC490)**.

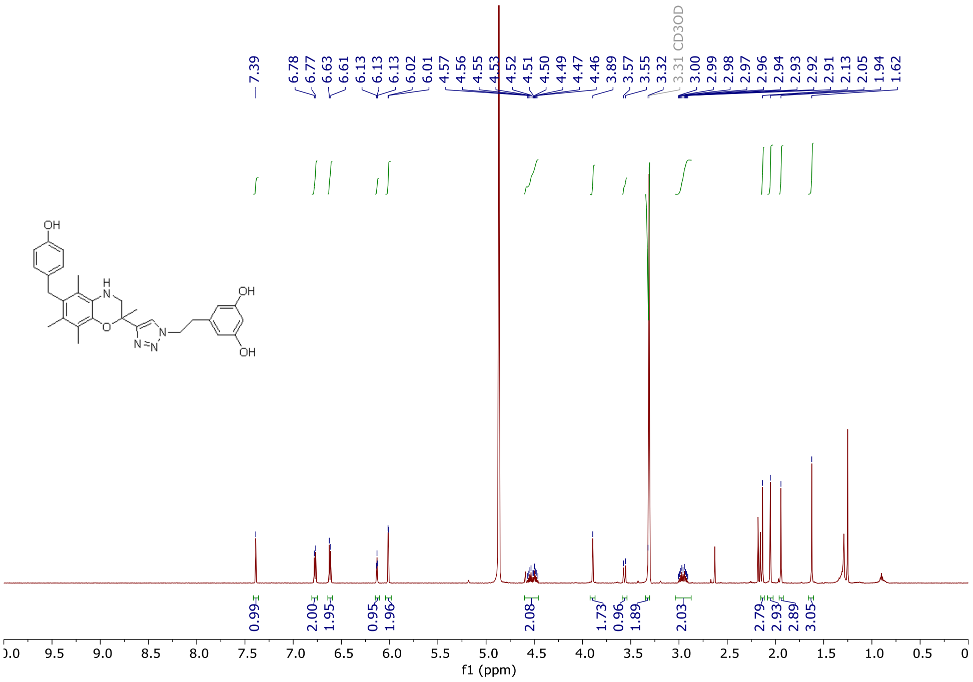

Figure S13. ¹H-NMR of compound **22a (TC491)**.

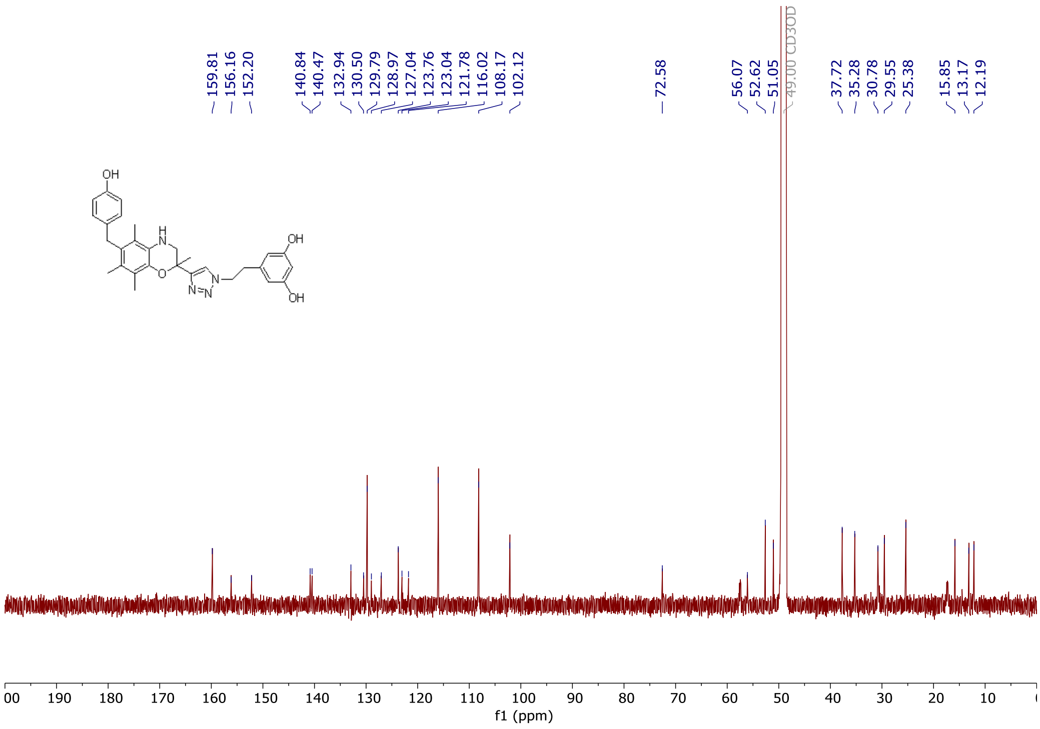

Figure S14. ¹³C-NMR of compound **22a (TC491)**.

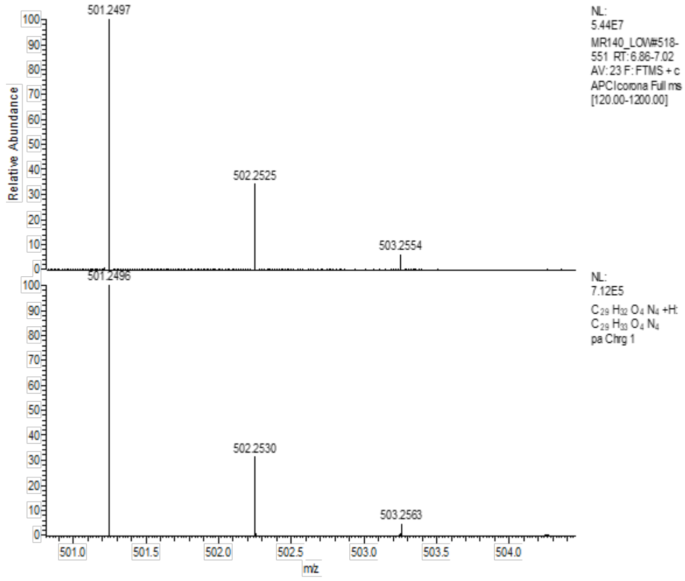

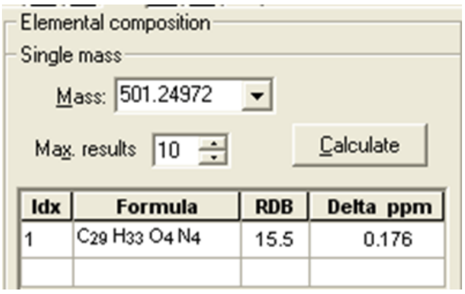
Elemental composition

Single mass

Mass: 501.24972

Max. results 10

Calculate

Idx	Formula	RDB	Delta ppm
1	$C_{29}H_{33}O_4N_4$	15.5	0.176

Figure S15. HRMS of compound **22a (TC491)**.

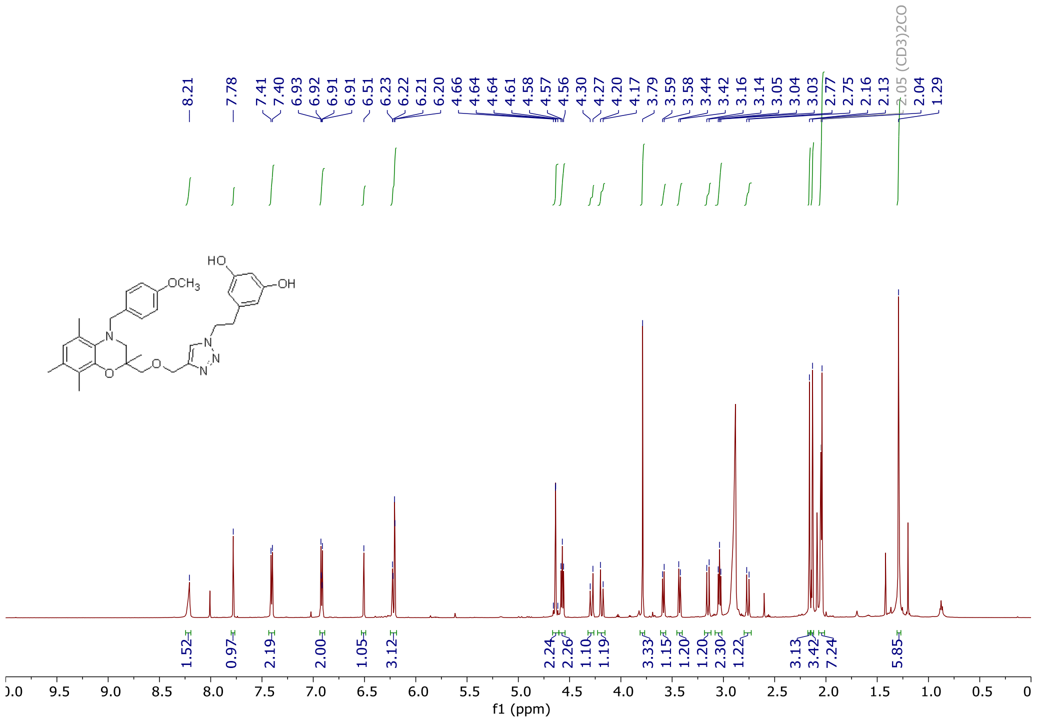

Figure S16. ^{1}H-NMR of compound **26 (TC484)**.

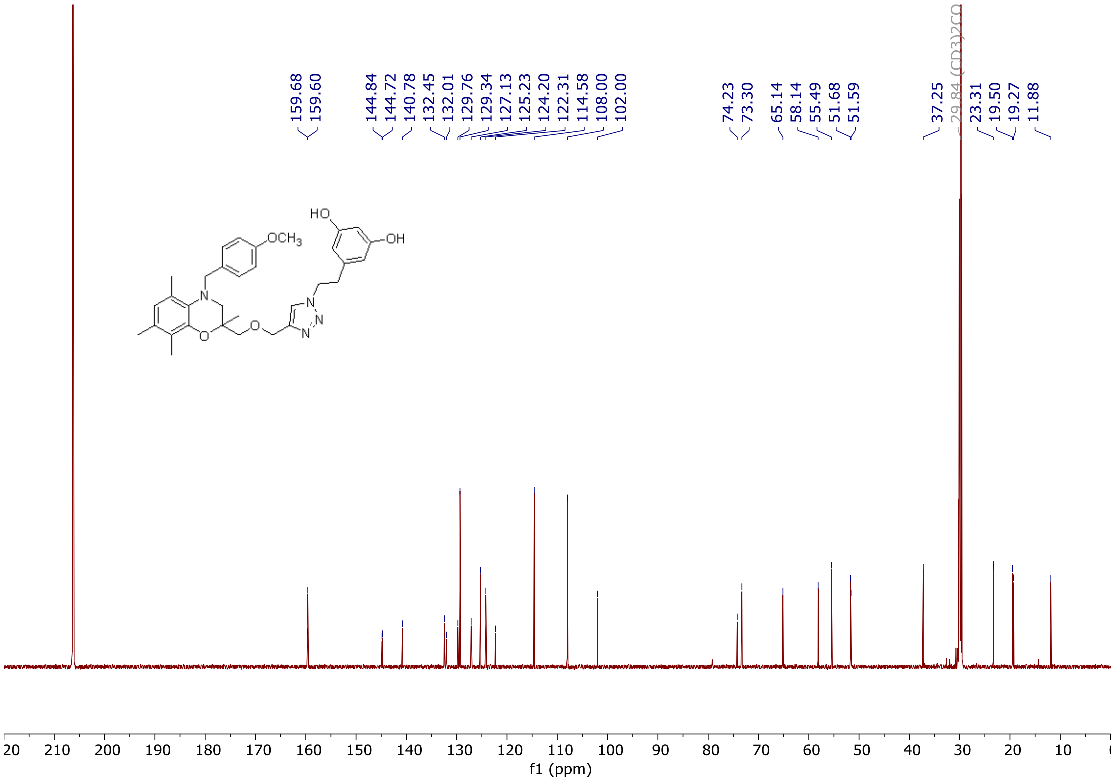

Figure S17. ^{13}C-NMR of compound **26 (TC484)**.

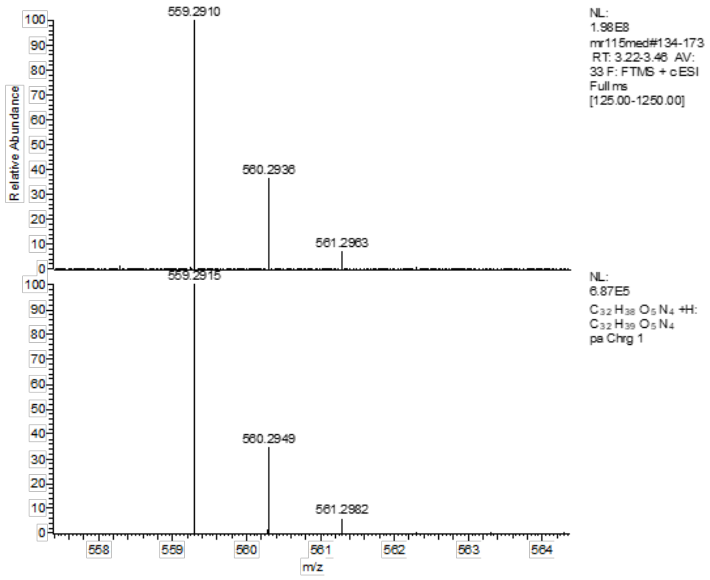

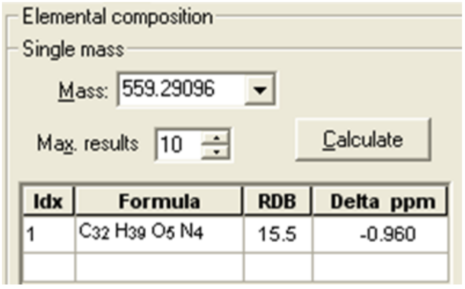
Elemental composition

Single mass

Mass: 559.29096

Max. results 10

Calculate

Idx	Formula	RDB	Delta ppm
1	$C_{32}H_{39}O_5N_4$	15.5	-0.960

Figure S18. HRMS of compound **26 (TC484)**.

3. Experimental Procedure for Neutral Red Assay

FF95 fibroblasts were plated in 96-well flat-bottomed black tissue culture-treated microplates in DMEM 10% FBS at a density of 10,000 cells/well. After an overnight incubation to ensure cell attachment, the test compounds were added at a final concentration of 10 μM and left to act on the cells for 72 hours. Subsequently, the medium was replaced by serum-free, phenol-red-free DMEM (PAN Biotech GmbH) containing neutral red (Sigma) at a final concentration of 0.0075% (w/v) for 4 h. Dye uptake by the viable cells was determined after dissolving with a solution of ethanol and water acidified with acetic acid. Neutral red fluorescence emission was measured at 645 nm following excitation at 530 nm in a Spark multimode microplate reader (Tecan Group Ltd, Männedorf, Switzerland). A corresponding vehicle (DMSO) dilution was used as control.

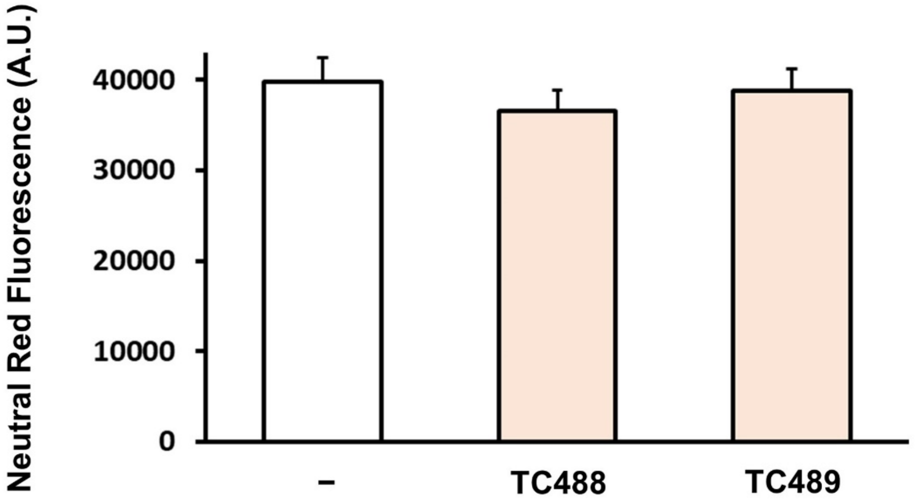

Figure S19. Cytotoxicity of the compounds **TC488** and **TC489** at 10 μM, as assessed with the Neutral Red method.
